# Ca^2+^ channels couple spiking to mitochondrial metabolism in substantia nigra dopaminergic neurons

**DOI:** 10.1126/sciadv.abp8701

**Published:** 2022-09-30

**Authors:** Enrico Zampese, David L. Wokosin, Patricia Gonzalez-Rodriguez, Jaime N. Guzman, Tatiana Tkatch, Jyothisri Kondapalli, William C. Surmeier, Karis B. D’Alessandro, Diego De Stefani, Rosario Rizzuto, Masamitsu Iino, Jeffery D. Molkentin, Navdeep S. Chandel, Paul T. Schumacker, D. James Surmeier

**Affiliations:** ^1^ Department of Neuroscience, Feinberg School of Medicine, Northwestern University, Chicago, IL 60611, USA.; ^2^ Aligning Science Across Parkinson’s (ASAP) Collaborative Research Network, Chevy Chase, MD 20815, USA.; ^3^ Department of Medicine, Feinberg School of Medicine, Northwestern University, Chicago, IL 60611, USA.; ^4^ Department of Biomedical Sciences, University of Padova, Padova 35131, Italy.; ^5^ Department of Physiology, Nihon University School of Medicine, 30-1, Oyaguchi Kami-cho, Itabashi-ku, Tokyo 173-8610, Japan.; ^6^ Department of Pediatrics, Cincinnati Children’s Hospital Medical Center, University of Cincinnati, Cincinnati, OH 45229, USA.; ^7^ Department of Pediatrics, Feinberg School of Medicine, Northwestern University, Chicago, IL 60611, USA.

## Abstract

How do neurons match generation of adenosine triphosphate by mitochondria to the bioenergetic demands of regenerative activity? Although the subject of speculation, this coupling is still poorly understood, particularly in neurons that are tonically active. To help fill this gap, pacemaking substantia nigra dopaminergic neurons were studied using a combination of optical, electrophysiological, and molecular approaches. In these neurons, spike-activated calcium (Ca^2+^) entry through Ca_v_1 channels triggered Ca^2+^ release from the endoplasmic reticulum, which stimulated mitochondrial oxidative phosphorylation through two complementary Ca^2+^-dependent mechanisms: one mediated by the mitochondrial uniporter and another by the malate-aspartate shuttle. Disrupting either mechanism impaired the ability of dopaminergic neurons to sustain spike activity. While this feedforward control helps dopaminergic neurons meet the bioenergetic demands associated with sustained spiking, it is also responsible for their elevated oxidant stress and possibly to their decline with aging and disease.

## Teaser

In SNc neurons, the opening of Ca^2+^ channels during spiking drives feedforward stimulation of mitochondrial ATP generation.

## INTRODUCTION

The brain is a bioenergetically demanding organ, accounting for roughly a fifth of the body’s total energy consumption (*1*). Much of this demand is attributable to regenerative “spike” activity in neurons that relies upon steep ionic gradients for Na^+^, K^+^, and Ca^2+^ across the plasma membrane that are maintained by adenosine triphosphate (ATP)–dependent pumps and transporters (*1*). Neurons also need ATP to drive a variety of spike-related processes, such as transmitter synthesis, storage, and release (*2*).

How neurons match the bioenergetic demands associated with spiking to the generation of ATP is poorly understood. In a behaving organism, neurons must be able not only to rapidly generate spikes in response to unpredictable synaptic inputs but also to sustain activity. If ATP production fails to keep pace with demand, spike generation becomes erratic or fails (*3*, *4*), compromising circuit function. In the course of evolution, the ionic mechanisms governing spiking appear to have been optimized to minimize bioenergetic cost within the constraints posed by the computational task a particular neuron needs to perform (*5*–*7*). Conversely, it is also likely that the mechanisms regulating ATP production have evolved to maximize the ability of neurons to fulfill their computational and circuit functions.

ATP generation in brain neurons relies upon a combination of glycolysis and mitochondrial oxidative phosphorylation (OXPHOS). It is generally thought that these linked bioenergetic cascades are governed by feedback mechanisms in which ATP is the controlled variable (*3*). Although glycolysis is capable of rapidly responding to ATP demand, it has a limited capacity (*2*, *8*, *9*). In contrast, mitochondrial OXPHOS has a greater ATP generation capacity but is slower to respond to alterations in cytosolic ATP levels (*10*, *11*). To minimize the potential ATP shortfall created by this lag, neurons have been hypothesized to engage an “anticipatory” feedforward control of mitochondrial OXPHOS where spike-triggered Ca^2+^ influx enters mitochondria to stimulate the generation of reducing equivalents for the electron transport chain (ETC) (*10*, *12*–*14*). However, the extent to which this type of mitochondrial control contributes to maintenance of cytosolic ATP levels during spiking, particularly sustained spiking, is uncertain, as are the molecular mechanisms that might mediate it.

To help fill this gap, a combination of electrophysiological, pharmacological, and imaging approaches was used to interrogate mouse substantia nigra pars compacta (SNc) dopaminergic neurons—neurons that manifest features consistent with feedforward mitochondrial control, such as spiking-associated cytosolic Ca^2+^ transients and mitochondrial oxidant stress (*15*). Genetically encoded, mitochondrially targeted, optical Ca^2+^ sensors, mice lacking the mitochondrial Ca^2+^ uniporter (MCU), and cytosolic sensors for the ratio of ATP to adenosine diphosphate (ADP) helped dissect mechanistic linkages (*16*–*18*). These studies suggest that spike-related opening of plasma membrane Ca_v_1 Ca^2+^ channels triggers Ca^2+^ release from the endoplasmic reticulum (ER) and feedforward stimulation of mitochondrial OXPHOS through two complementary pathways, which enable these neurons to sustain spiking for extended periods. Our findings also provide a context for understanding the selective vulnerability of these neurons to aging and neurodegenerative diseases with mitochondrial determinants, like Parkinson’s disease (PD).

## RESULTS

### Ca_v_1 Ca^2+^ channels regulate ryanodine receptor–dependent ER Ca^2+^ release

**Fig. 1.**
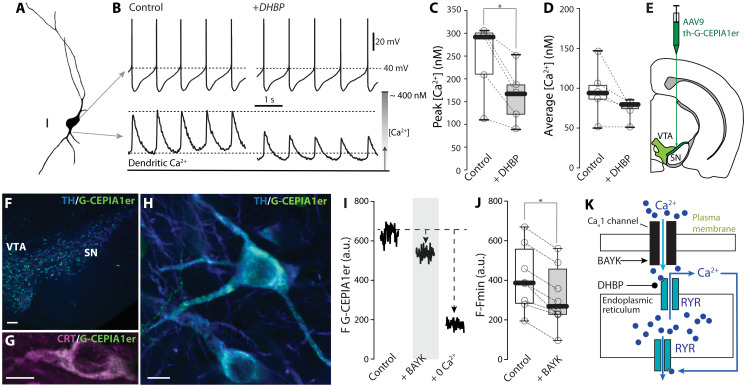
Ca^2+^ entry through Ca_v_1 Ca^2+^ channels triggered ER Ca^2+^ release. (**A**) Representative reconstruction of a Fura-2–filled SNc dopaminergic neuron (scale bar, 20 μm). (**B**) Representative whole-cell current clamp recordings (top) and 2PLSM traces of cytosolic Ca^2+^ oscillations (bottom) in SNc neurons loaded with Fura-2, before and after application of RYR antagonist DHBP. (**C** and **D**) Box plots summarizing the average amplitude of cytosolic Ca^2+^ oscillations and average Ca^2+^ concentration in dopaminergic neurons in control conditions and after DHBP bath application; DHBP significantly decreased the amplitude of cytosolic Ca^2+^ oscillations (*n* = 5, *N* = 5; *P* = 0.0312, one-tailed Wilcoxon matched-pairs signed rank test). (**E**) Cartoon representing the AAV delivery strategy to induce expression of G-CEPIA1er in midbrain dopaminergic neurons; modified from the Allen Mouse Brain Atlas, online version 1, 2008 (https://atlas.brain-map.org/). (**F** to **H**) Immunofluorescence images showing the expression of G-CEPIA1er in dopaminergic neurons stained for TH and the colocalization of G-CEPIA1er with the ER marker calreticulin (CRT); scale bars, 100 μm (F) and 10 μm (G and H). (**I**) Representative 2PLSM imaging time series showing the effect of application of Ca_v_1 Ca^2+^ channel (Ca_v_1)–positive allosteric modulator Bay K8644 (BAYK) on dendritic ER Ca^2+^ in SNc dopaminergic neurons expressing G-CEPIA1er. (**J**) Quantification of the effect of BAYK on dendritic ER Ca^2+^ measured with G-CEPIA1er (*n* = 8, *N* = 7; *P* = 0.0039, one-tailed Wilcoxon matched-pairs signed rank test). (**K**) Cartoon illustrating the effect of Ca^2+^ entry through Ca_v_1 channels on RYR Ca^2+^ release and the targets of BAYK and DHBP. Box plots indicate first and third quartiles, thick center lines represent medians, and whiskers indicate the range. a.u., arbitrary units. *P < 0.05.

Cytosolic Ca^2+^ oscillations in SNc dopaminergic neurons are dependent on opening of plasma membrane Ca_v_1 Ca^2+^ channels (*19*). In other cell types, Ca_v_1 channels are physically close or coupled to ryanodine receptors (RYRs) positioned in the ER (*20*–*22*). Because RYRs are allosterically modulated by cytosolic Ca^2+^ (*23*), this coupling allows Ca^2+^ entry through Ca_v_1 channels to trigger Ca^2+^-induced Ca^2+^ release (CICR) from the ER, which propagates away from the surface to intracellular locations (*24*). To determine whether a similar mechanism was engaged by Ca_v_1 channels during pacemaking, SNc neurons were patched in ex vivo brain slices and filled with the Ca^2+^ indicator Fura-2 (Fig. 1A); dendritic fluctuations in Fura-2 fluorescence were measured using two-photon laser scanning microscopy (2PLSM) and aligned with changes in transmembrane voltage during pacemaking (Fig. 1B) (*25*). To determine whether RYR-dependent CICR contributed to the cytosolic Ca^2+^ oscillation, RYRs were inhibited by bath application of 1,1′-diheptyl-4,4′-bipyridinium dibromide (DHBP; 100 μM) (*26*). Inhibiting RYRs significantly reduced the amplitude of cytosolic Ca^2+^ transients time-locked to pacemaking (Fig. 1, B to D).

To further explore the coupling of Ca_v_1 channels to ER Ca^2+^ stores, an adeno-associated virus (AAV) carrying an expression construct in which a tyrosine hydroxylase (TH) promoter fragment controlled the expression of an ER-targeted Ca^2+^ sensor (G-CEPIA1er) (*27*) was stereotaxically injected into the SNc (Fig. 1, E to H). In ex vivo brain slices taken from these mice several weeks later, 2PLSM was used to monitor changes in G-CEPIA1er fluorescence. As expected, inhibiting the sarco/endoplasmic reticulum Ca^2+^–adenosine triphosphatase (ATPase) (SERCA) with cyclopiazonic acid (20 μM) decreased G-CEPIA1er fluorescence, showing that the probe was capable of signaling depletion of ER Ca^2+^ (fig. S1). If the opening of Ca_v_1 channels was prolonged with the positive allosteric modulator BAYK-8644 (1 μM), dendritic ER Ca^2+^ fell, confirming the coupling of Ca_v_1 Ca^2+^ channels and the ER in SNc dopaminergic neurons (Fig. 1, I to K).

### RYR-dependent Ca^2+^ release increased mitochondrial matrix Ca^2+^

**Fig. 2.**
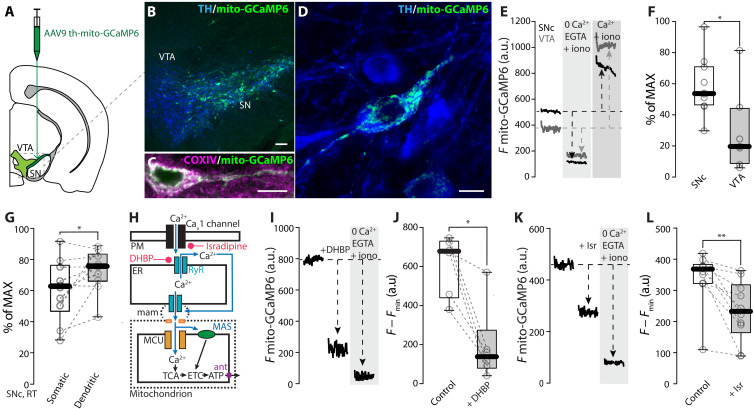
RYR and Ca_v_1 channels determine elevated intra-mitochondrial Ca^2+^ in SNc dopaminergic neurons. (**A**) Cartoon representing the AAV delivery strategy to express mito-GCaMP6 in midbrain dopaminergic neurons; modified from the Allen Mouse Brain Atlas, online version 1, 2008 (https://atlas.brain-map.org/). (**B** to **D**) Immunofluorescence images showing the expression of mito-GCaMP6 in dopaminergic neurons stained for TH and the colocalization of mito-GCaMP6 with the mitochondrial marker COXIV; scale bars, 100 μm (B) and 10 μm (C and D). (**E**) Representative 2PLSM imaging time series showing the estimation of baseline mitochondrial Ca^2+^ levels in SNc (black) and VTA (gray) dopaminergic neurons expressing mito-GCaMP6. The Ca^2+^ ionophore ionomycin (iono) is used to allow the movement of Ca^2+^ through membranes according to its concentration gradient. (**F**) Mitochondrial Ca^2+^ levels in SNc and VTA dopaminergic neurons estimated with mito-GCaMP6 (*n* = 10, *N* = 10; *n* = 7, *N* = 7 for SNc and VTA, respectively; *P* = 0.0136, two-tailed Mann-Whitney test). (**G**) Dendritic and somatic mitochondrial Ca^2+^ levels in SNc dopaminergic neurons estimated with mito-GCaMP6 (*n* = 10, *N* = 9; *P* = 0.0273, two-tailed Wilcoxon matched-pairs signed rank test). (**H**) Cartoon illustrating the effect of Ca^2+^ entry through Ca_v_1 channels and RYR Ca^2+^ release on mitochondrial Ca^2+^ loading and the targets of isradipine and DHBP. (**I**) Representative 2PLSM imaging time series showing the effect of application of DHBP on mitochondrial Ca^2+^ in SNc neurons. (**J**) Effect of DHBP (100 μM) on mitochondrial Ca^2+^ measured with mito-GCaMP6 (*n* = 6, *N* = 4; *P* = 0.0156, one-tailed Wilcoxon matched-pairs signed rank test). (**K**) Representative 2PLSM imaging time series showing the effect of application of Ca_v_1-negative allosteric modulator isradipine (Isr) on mitochondrial Ca^2+^ in SNc neurons. (**L**) Effect of isradipine (0.5 to 1 μM) on mitochondrial Ca^2+^ measured with mito-GCaMP6 (*n* = 10, *N* = 9; *P* = 0.0010, one-tailed Wilcoxon matched-pairs signed rank test). Box plots indicate first and third quartiles, thick center lines represent medians, and whiskers indicate the range. *P < 0.05, **P < 0.01.

Mitochondrial Ca^2+^ uptake occurs preferentially at sites of juxtaposition with the ER [mitochondria-associated membranes (MAMs)], where Ca^2+^ released from RYRs can reach concentrations required to gate the MCU (*28*). To monitor mitochondrial matrix Ca^2+^ concentration during pacemaking, a Ca^2+^ sensor targeted to the mitochondrial matrix (mito-GCaMP6) was used (*16*, *29*). Following mesencephalic injection of an AAV carrying a TH-controlled mito-GCaMP6 expression construct, fluorescence was found throughout the SNc and was colocalized with the mitochondrial marker COXIV (cytochrome c oxidase subunit 4) (Fig. 2, A to D). 2PLSM was used to monitor mitochondrial matrix Ca^2+^ in SNc dopaminergic neurons in ex vivo brain slices. Using a simple calibration procedure (see Materials and Methods; fig. S2, A and B), an estimate of matrix Ca^2+^ level was made in SNc and ventral tegmental area (VTA) dopaminergic neurons. This value was greater in SNc dopaminergic neurons than those in the VTA (Fig. 2, E and F). Moreover, mitochondrial matrix Ca^2+^ concentration was higher for mitochondria in dendrites than those in the somatic region (Fig. 2G and fig. S2, C and D).

To assess the role of Ca_v_1 channels and RYRs in determining matrix Ca^2+^ levels, pharmacological tools were used (Fig. 2H). Bath application of DHBP (50 to 100 μM) reduced mitochondrial Ca^2+^ levels in SNc dopaminergic neurons (Fig. 2, I and J). Similarly, bath application of the Ca_v_1 channel negative allosteric modulator (NAM) isradipine (0.5 to 1 μM) significantly decreased mitochondrial Ca^2+^ levels (Fig. 2, K and L). Together, these experiments establish a mechanistic linkage between plasma membrane Ca_v_1 channels, RYRs, and mitochondrial matrix Ca^2+^ concentration.

Another ER Ca^2+^-release channel enriched at MAMs is the inositol triphosphate (IP_3_) receptor (IP_3_R), which is gated by IP_3_ generated by phospholipase C (PLC) stimulated by metabotropic receptors, like type I metabotropic glutamate receptors [mGluR-I; (*30*)]. Bath application of the mGluR-I agonist (*S*)-3,5-dihydroxyphenylglycine [DHPG; 10 μM; (*31*)] induced a rapid increase in intramitochondrial Ca^2+^ levels in SNc dopaminergic neurons (fig. S2, E and F). Thus, both RYRs and IP_3_Rs can be engaged to elevate matrix Ca^2+^ levels in pacemaking SNc dopaminergic neurons.

### Mitochondrial matrix Ca^2+^ tracks spike rate

**Fig. 3.**
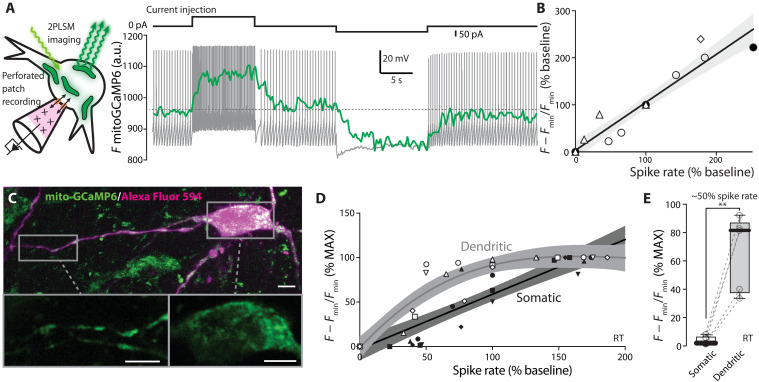
Mitochondrial matrix Ca^2+^ tracks spike rate. (**A**) Left: Cartoon representing the strategy to monitor mitochondrial Ca^2+^ dynamics during changes in firing frequency via perforated-patch recording configuration. Right: Representative experiment illustrating the electrophysiological recording (gray, dotted line indicates −45 mV) and mito-GCaMP6 trace (green) during current injection. (**B**) Plot illustrating the distribution of normalized mito-GCaMP6 fluorescence over the normalized spike rate in SNc dopaminergic neurons (linear fit and 95% confidence interval, *n* = 4, *N* = 4). (**C**) 2PLSM image of an SNc dopaminergic neuron expressing mito-GCaMP6 and filled with Alexa Fluor 594 dye after inducing break-in at the end of a perforated-patch recording coupled with mito-GCaMP6 imaging in the somatic and dendritic regions, highlighted in the insets (scale bars: 50 μm, main image; 10 μm, insets). (**D**) Plot illustrating the distribution of normalized mito-GCaMP6 fluorescence over the normalized spike rate for dendritic (gray, empty symbols) and somatic (black, filled symbols) mitochondria, within the same SNc dopaminergic neurons (empty/filled symbols of the same shape indicate paired measurements from the same neuron; *n* = 5, *N* = 5; soma: linear fit and 95% confidence interval; dendritic measurements were better fitted by a third-order polynomial fit and 95% confidence interval). (**E**) Distribution of the normalized mito-GCaMP6 fluorescence in dendritic and somatic mitochondria at 50% of the baseline spike rate (*n* = 5, *N* = 5; *P* = 0.0050, two-tailed paired *t* test). Box plots indicate first and third quartiles, thick center lines represent medians, and whiskers indicate the range. **P < 0.01.

SNc dopaminergic neurons are autonomous pacemakers, spiking regularly at low rates in the absence of synaptic input (*32*, *33*). This basal discharge rate is modulated up and down by excitatory and inhibitory synapses, respectively (*34*). Plasma membrane Ca_v_1 Ca^2+^ channels provide a continuous readout of membrane depolarization and spiking that could be used to stimulate feedforward control of mitochondrial OXPHOS. As a first step toward testing this hypothesis, the membrane potential of SNc dopaminergic neurons was monitored and manipulated using the perforated-patch recording method in ex vivo brain slices while monitoring mitochondrial matrix Ca^2+^ using mito-GCaMP6 (as above). The perforated-patch configuration only allows monovalent cations to pass from the electrode to the cell, preserving intracellular Ca^2+^ signaling and metabolism (Fig. 3A, left). In these experiments, mito-GCaMP6 fluorescence was continuously monitored while slowing or accelerating spiking with intracellular current injection (Fig. 3A, right). Mitochondrial Ca^2+^ levels (as measured by mito-GCaMP6 fluorescence) tracked spike rate: decreasing as spiking slowed and increasing as spiking accelerated (Fig. 3A, right). After calibration, the somatic mito-GCaMP6 signal was plotted as a function of spike rate. Astonishingly, these data were well fit with a straight line (Fig. 3B).

As shown above, the Ca^2+^ concentration in the matrix of dendritic mitochondria was higher than that in the matrix of somatic mitochondria in the absence of stimulation (Fig. 2G). As the dendrites of SNc dopaminergic neurons faithfully backpropagate initial segment spikes (*35*, *36*), dendritic mitochondria should increase their Ca^2+^ loading with spiking. To test this hypothesis, the experiments described above were repeated while monitoring the mito-GCaMP6 signal in dendritic and somatic mitochondria (Fig. 3C). The Ca^2+^ content of the matrix in dendritic mitochondria exhibited a greater sensitivity to low-frequency spiking than did somatic mitochondria (Fig. 3, D and E). Increasing the spike rate further led to sublinear increments in the dendritic mito-GCaMP6 fluorescence, presumably reaching the limit of the probe’s dynamic range.

### Matrix Ca^2+^ loading stimulates mitochondrial OXPHOS

**Fig. 4.**
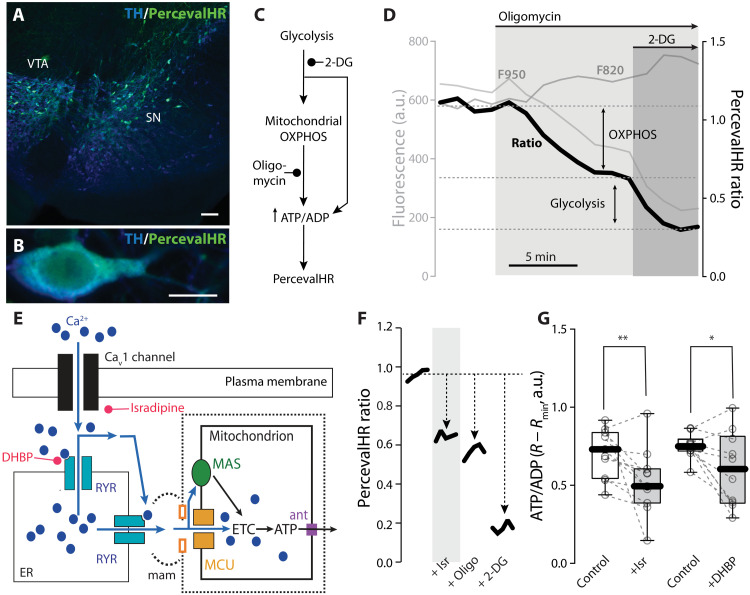
The Ca_v_1-RYR couple drives mitochondrial OXPHOS. (**A** and **B**) Immunofluorescence images showing the expression of PercevalHR in dopaminergic neurons stained for TH; scale bars, 100 μm (A) and 10 μm (B). (**C**) Schematic representing the contribution of glycolysis and mitochondrial OXPHOS to the production of ATP and maintenance of the ATP/ADP ratio, measured with PercevalHR; the actions of 2-DG and oligomycin are indicated. (**D**) Representative PercevalHR experiment estimating the contribution of mitochondria (inhibited by oligomycin) and glycolysis (inhibited by 2-DG) to the ATP/ADP ratio of the cell; the fluorescence measured for each of the two wavelengths (950 for ATP, 820 for ADP; gray traces) and the F950/F820 ratio calculated for each time point (black trace) are illustrated. (**E**) Cartoon illustrating the effect of Ca^2+^ entry through Ca_v_1 channels and RYR Ca^2+^ release on mitochondrial ATP production and the action of isradipine and DHBP. (**F**) Representative 2PLSM ratio imaging time series showing the effect of application of Ca_v_1 antagonist isradipine (Isr; 1 μM), followed by oligomycin (Oligo; 10 μM) and substitution of glucose with 2-DG on PercevalHR ratio in SNc dopaminergic neurons expressing PercevalHR. (**G**) Quantification of the effect of isradipine (1 μM) and DHBP (100 μM) on ATP/ADP ratio in SNc neurons expressing PercevalHR (isradipine, *n* = 11, *N* = 8; *P* = 0.0049, one-tailed Wilcoxon matched-pairs signed rank test; DHBP, *n* = 10, *N* = 6; *P* = 0.032, one-tailed Wilcoxon matched-pairs signed rank test). Box plots indicate first and third quartiles, thick center lines represent medians, and whiskers indicate the range. *P < 0.05, **P < 0.01.

Ca^2+^ entry into the mitochondrial matrix is well known to activate tricarboxylic acid cycle (TCA) enzymes and increase the production of reducing equivalents for the ETC (*37*); doing so increases the capacity of mitochondrial complex V (MCV or ATP synthase) to convert ADP to ATP (*38*). To test the hypothesis that Ca_v_1 channel/RYR–mediated mitochondrial Ca^2+^ loading was driving ATP production during spiking, the cytosolic ATP/ADP ratio needed to be monitored. To this end, AAVs carrying a TH-driven expression construct for the ratiometric ATP/ADP probe PercevalHR were stereotaxically injected into the SN of mice (*17*, *39*). Two weeks later, mice were sacrificed and ex vivo brain slices were prepared for 2PLSM interrogation (Fig. 4, A and B). Bath application of oligomycin (10 μM) was used to inhibit MCV, and the nonhydrolyzable glucose analog 2-deoxy-glucose (2-DG; 3.5 mM) was substituted for glucose to disrupt glycolysis (Fig. 4, C and D). At rest (when SNc dopaminergic neurons are pacemaking at a slow rate), the relative contribution of mitochondrial OXPHOS to maintenance of the cytosolic ATP/ADP ratio (“OXPHOS index”) was high (~70%). Elevating extracellular glucose decreased this contribution, as glycolysis accounted for a larger fraction of ATP production (Fig. 4D and fig. S3A).

Next, the contribution of Ca_v_1 channels and RYRs to maintenance of cytosolic ATP/ADP ratio was determined (Fig. 4, E to G). Bath application of isradipine (1 μM) to inhibit Ca_v_1 channels significantly decreased the PercevalHR ratio (Fig. 4, F and G). This change was specific to the oligomycin-sensitive PercevalHR signal, arguing that it was mitochondrial in origin (fig. S3, B and C). Bath application of DHBP (100 μM) to inhibit RYRs had a very similar effect on the estimated ATP/ADP ratio, and the response to oligomycin (Fig. 4G and fig. S3, B and C). Thus, in SNc dopaminergic neurons, the Ca_v_1 channel/RYR couple specifically stimulated mitochondrial OXPHOS and ATP production.

### Feedforward stimulation of mitochondria is necessary to sustain elevated spike rates

**Fig. 5.**
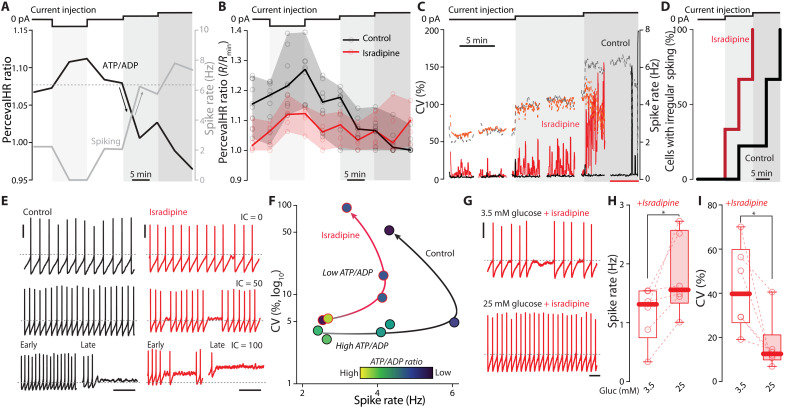
Feedforward stimulation of mitochondria by Ca^2+^ is required to sustain firing. (**A**) Representative perforated-patch and PercevalHR 2PLSM imaging experiment in SNc dopaminergic neurons modulating spike rate with current injections; spike rate, gray; ATP/ADP, black; current depolarizing steps (top), 50 pA. (**B**) Quantification of PercevalHR ratio upon changes in spike rate as in (A) for control (black) and isradipine-treated (0.5 μM, red) neurons; medians, range, and individual points are depicted (controls: *n* = 9, *N* = 9; isradipine: *n* = 6, *N* = 6). (**C**) Representative plots of instantaneous CV (continuous traces) and spike rate (dashed traces) for control (black) and isradipine-treated (red) cells during the depolarization steps [see (A)]. (**D**) Cumulative probability plots of cells undergoing failure to sustain firing at different time points for control (black) and isradipine-treated (red) SNc neurons during the current injection protocol in (A) (control: *n* = 9, *N* = 9; isradipine: *n* = 6, *N* = 6; *P* = 0.0028, Mantel-Cox test). (**E**) Representative recordings for control (black) and isradipine-treated (red) dopaminergic neurons during the depolarization steps. (**F**) Representative plots showing the correlation between spike rate (*x* axis), CV (*y* axis, logarithmic scale), and ATP/ADP ratio (colorimetric scale) for control and isradipine-treated cells; the colorimetric scale indicates the maximum (yellow), the minimum (dark purple), and the intermediate ratio values recorded within each experiment; see fig. S5 for details. (**G**) Representative recordings of isradipine-treated SNc neurons in standard glucose (3.5 mM) aCSF and 25 mM glucose aCSF. (**H**) Effect of glucose concentration on spike rate of SNc isradipine-treated neurons (*n* = 6, *N* = 5; *P* = 0.0156, one-tailed Wilcoxon matched-pairs signed rank test). (**I**) Effect of increasing glucose concentration on the CV of isradipine-treated neurons (*n* = 6, *N* = 5; *P* = 0.0156, one-tailed Wilcoxon matched-pairs signed rank test). Scale bars (E and G), 20 mV, 1 s. Box plots indicate first and third quartiles, thick center lines represent medians, and whiskers indicate the range. *P < 0.05.

One of the principal bioenergetic challenges facing neurons is to supply the ATP necessary for efficient operation of plasma membrane pumps and transporters responsible for maintaining ionic gradients required for spiking (*3*, *40*). Failing to keep up with the ATP demand can lead to depolarization, persistent Na_v_1 Na^+^ channel inactivation, and an inability to spike (*3*). To determine whether cytosolic ATP/ADP ratio tracked spiking in SNc dopaminergic neurons, they were induced to express PercevalHR and then studied using a combination of 2PLSM and perforated-patch methods in ex vivo brain slices (as described above). Spiking rate was modulated by 10-min-long somatic current steps (50 pA), and ATP/ADP ratio was assessed every 5 min. As expected from the experiments monitoring mitochondrial matrix Ca^2+^ concentration during similar perturbations, the cytosolic ATP/ADP ratio closely tracked spike rate over these periods, rising when cells were silenced and then falling progressively as the spiking rate increased (Fig. 5A). To assess the role of feedforward control of mitochondria in regulating ATP/ADP ratios, slices were preincubated and maintained in isradipine (0.5 μM) during perforated-patch recording. Consistent with a stimulatory role, inhibition of Ca_v_1 channels decreased the basal ATP/ADP ratio (Fig. 5B). With increased stimulation, the ATP/ADP ratio fell in both control and isradipine-treated cells, approaching an asymptote with high levels of current injection (Fig. 5B).

Examination of the activity evoked by current injection revealed why there was a convergence in ATP/ADP ratios. As shown previously (*19*), isradipine preincubation had no effect on pacemaking rate; however, Ca_v_1 channel inhibition increased the irregularity of spiking at baseline, leading to an increase in the interspike interval coefficient of variation (CV) (Fig. 5, C to E). In isradipine-treated neurons, the irregularity in spiking paralleled the drop in ATP/ADP ratio until spiking stopped entirely; in contrast, when feedforward control was intact, the regularity in spiking was maintained until late in the intervals producing the highest spiking rates (Fig. 5, C to E). As expected from previous modeling results (*3*), when neurons were forced to rely upon feedback control of mitochondrial OXPHOS in the presence of isradipine, the average membrane potential became progressively more depolarized until they stopped spiking and stayed at depolarized membrane potentials, suggesting that Na^+^/K^+^ pumps were falling behind the demand created by channel opening and this led to depolarization block. Consistent with the impact of progressive depolarization on the inactivation of voltage-dependent Na^+^ channels driving spiking, as irregularity in spiking increased, the spike threshold rose and the rate of rise of the spike (*dV*/*dt*) fell (fig. S4). To better illustrate the relationship between spiking and ATP/ADP ratio in SNc dopaminergic neurons, the plots of CV as a function of spike rate were generated for control and isradipine-treated neurons in which the ATP/ADP ratio was color-coded (Fig. 5F and fig. S5). These plots show that a declining ATP/ADP ratio was correlated with irregular spiking in both conditions, but that in isradipine-treated neurons even modest elevations in spike rate pushed down this ratio, leading to increased irregularity and ultimately depolarization block.

A potential complication in the interpretation of these results is that one subtype of Ca_v_1 channel expressed by SNc dopaminergic neurons—those with a Ca_v_1.3 pore-forming subunit—might be supporting regenerative activity by giving rise to a depolarizing inward current at subthreshold membrane potentials (*41*, *42*). If this function of Ca_v_1.3 channels—rather than their control of bioenergetics—was the principal factor determining the impact of isradipine on spiking rate and regularity, then increasing glycolytic ATP production by elevating extracellular glucose concentration should have little or no effect on spiking in the presence of isradipine. However, this was not the case. In the presence of isradipine and physiological concentrations of glucose (3.5 mM), spiking was slow and irregular, as shown above. Boosting extracellular glucose to 25 mM increased spiking rate and reduced irregularity (Fig. 5, G to I). Thus, the principal effect of inhibiting Ca_v_1 channels on repetitive spiking appears to be mediated by a bioenergetic, not an electrogenic, deficit.

### Conditional deletion of the MCU increased reliance upon MAS

**Fig. 6.**
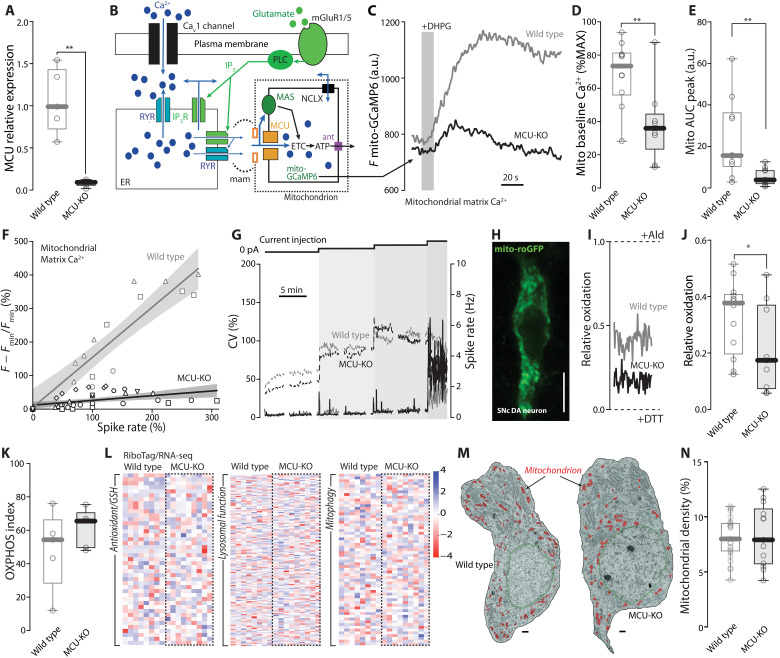
Loss of MCU disrupts mitochondrial Ca^2+^ uptake but results in a modest phenotype. (**A**) Quantification of the relative expression of *Mcu* mRNA in SNc dopaminergic neurons collected with the RiboTag strategy from wild-type and MCU-KO mice (*N* = 5 and 6 for wild type and MCU-KO, respectively; *P* = 0.0022, one-tailed Mann-Whitney test). (**B**) Cartoon illustrating the pathways leading to mitochondrial Ca^2+^ uptake tested in wild-type and MCU-KO neurons expressing mito-GCaMP6: Ca^2+^ entry through Ca_v_1 triggers gates RYRs and release of Ca^2+^ at the “mitochondria-associated membrane” (mam); stimulation of mGluRs coupled to PLC generates IP_3_, that gates IP_3_R and induces release of Ca^2+^ at the mam; the MCU complex is gated by high Ca^2+^ microdomains and allows accumulation of Ca^2+^ into the mitochondrial matrix. (**C**) Representative 2PLSM mito-GCaMP6 traces of wild-type (gray) and MCU-KO (black) SNc dopaminergic neurons stimulated with the mGluR-I agonist DHPG (10 μM). (**D**) Quantification of mitochondrial baseline Ca^2+^ in wild-type and MCU-KO dopaminergic neurons expressing mito-GCaMP6 (*n* = 10, *N* = 9; *n* = 9, *N* = 8 for wild-type and MCU-KO, respectively; *P* = 0.0086, one-tailed Mann Whitney test). (**E**) Quantification of mitochondrial Ca^2+^ uptake upon DHPG stimulation in wild-type and MCU-KO SNc neurons expressing mito-GCaMP6; the area under the curve (AUC) is calculated over 90 s from the start of the peak (*n* = 10, *N* = 9 for wild type; *n* = 10, *N* = 9 for MCU-KO; *P* = 0.0014, one-tailed Mann Whitney test). (**F**) Plot illustrating the distribution of normalized mito-GCaMP6 fluorescence over the normalized spike rate in wild-type (gray) and MCU-KO (black) SNc dopaminergic neurons (linear fit and 95% confidence interval, *n* = 3, *N* = 2 for wild type; *n* = 6, *N* = 6 for MCU-KO). (**G**) Representative plots of instantaneous CV (continuous traces) and spike rate (dashed traces) for control (gray) and MCU-KO (black) SNc dopaminergic neurons during depolarization steps. (**H**) Representative 2PLSM image of an SNc dopaminergic neuron expressing mito-roGFP (scale bar, 10 μm). (**I**) Representative 2PLSM mito-roGFP measurements for wild-type (gray) and MCU-KO (black) SNc neurons expressed as relative oxidation compared to the fully reduced and fully oxidized states obtained upon application of dithiothreitol (DTT; 2 mM) and aldrithiol (Ald; 200 μM), indicated by dashed lines. (**J**) Quantification of the relative mitochondrial oxidation in wild-type and MCU-KO SNc neurons (*n* = 12, *N* = 9 for wild type; *n* = 9, *N* = 6 for MCU-KO; *P* = 0.047, one-tailed Mann-Whitney test). (**K**) Quantification of the OXPHOS index estimated in wild-type and MCU-KO SNc neurons expressing PercevalHR (*n* = 5, *N* = 4 for wild type; *n* = 5, *N* = 4 for MCU-KO). (**L**) Heatmaps of differential expression of genes linked to the antioxidant defense and glutathione (GSH) synthesis, lysosomal function, and mitophagy obtained from RNA-seq analysis from wild-type and MCU-KO SNc neurons expressing RiboTag (*N* = 8 and 9 for wild type and MCU-KO, respectively). (**M**) Electron micrographs of identified SNc dopaminergic neurons from wild-type and MCU-KO mice; nuclear membrane is outlined in green, mitochondria are labeled in red; scale bar, 1 μm. (**N**) Box plots showing no difference in mitochondrial density in wild-type and MCU-KO neurons (*n* = 23, *N* = 2; *n* = 13, *N* = 2, respectively). Box plots indicate first and third quartiles, thick center lines represent medians, and whiskers indicate the range. *P < 0.05, **P < 0.01.

Ca^2+^ release by the ER can affect mitochondrial OXPHOS in two ways (*14*, *43*). The best described of these is mediated by Ca^2+^ flux into the mitochondrial matrix through the MCU (*43*–*45*). To assess the importance of this pathway, mice in which the gene coding for the MCU was floxed (*18*) were crossed with mice expressing Cre recombinase under the control of the dopamine transporter (DAT). The conditional knockout (KO) of the MCU in these mice was verified by isolating mRNA specifically from dopaminergic neurons using the RiboTag method (*39*, *46*) and then performing a quantitative reverse transcriptase polymerase chain reaction (qRT-PCR) assay targeting MCU transcripts (Fig. 6A). The efficacy of the conditional KO was also verified functionally by monitoring mitochondrial matrix Ca^2+^ in SNc dopaminergic neurons expressing mito-GCaMP6 after stimulation of mGluRs with DHPG (10 μM) (Fig. 6, B and C). At baseline, matrix Ca^2+^ levels were lower in MCU-KOs than in wild-type neurons and the response to DHPG stimulation was significantly less than in the wild type (Fig. 6, C to E). Moreover, increasing spike rate in MCU-KO neurons using perforated-patch methods failed to elevate mitochondrial matrix Ca^2+^ concentration as described above (Fig. 6F and fig. S6).

Despite the loss of Ca^2+^ coupling to the mitochondrial matrix, there were few alterations in cytosolic Ca^2+^ handling, spiking, and gene expression in SNc dopaminergic neurons lacking the MCU. For example, cytosolic Ca^2+^ transients evoked by DHPG were not affected in MCU-KO neurons (fig. S7, A to C). Wild-type and MCU-KO neurons responded to current injection with perforated-patch electrodes in a very similar way (Fig. 6G and fig. S7, D to H). Moreover, although mitochondrial oxidant stress in MCU-KO SNc dopaminergic neurons measured with the genetically encoded sensor mito-roGFP (reduction-oxidation–sensitive green fluorescent protein) was modestly lower than in wild-type neurons (Fig. 6, H to J), the OXPHOS index in MCU-KO neurons estimated with PercevalHR was similar to that in wild-type neurons (Fig. 6K). RNA sequencing (RNA-seq) analysis failed to reveal changes in expression of genes linked to Ca^2+^ homeostasis (fig. S8), pacemaking, metabolism, or dopaminergic neurotransmission (fig. S9A) and indicated only modest changes in the expression of genes linked to oxidant defenses, lysosomes, and mitophagy (Fig. 6L). Moreover, histological inspection of TH immunoreactivity in the striatum and mesencephalon revealed no differences from wild-type controls (fig. S9, B to E), and transmission electron micrographs found no differences in mitochondrial morphology or density at the cell body level (Fig. 6, M and N). Last, MCU-KO mice were normal in assays of open field behavior, gait, and fine motor skills (fig. S9, F to K).

### What accounts for the relative insensitivity of SNc dopaminergic neurons to MCU deletion?

**Fig. 7.**
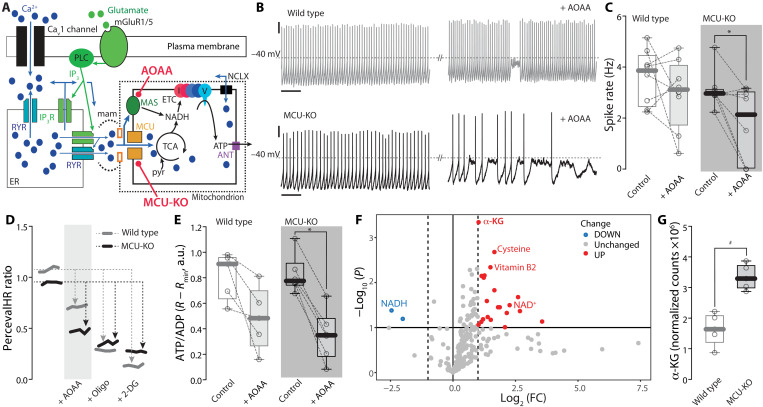
In MCU-KO neurons, firing is sustained by increased reliance on MAS. (**A**) Cartoon illustrating the pathways through which Ca^2+^ regulates mitochondrial ATP production, either by entering the mitochondrial matrix via MCU and stimulating the TCA cycle or by stimulating the MAS, which can transfer cytosolic reducing equivalents into the mitochondria, where they can be used to fuel OXPHOS; the target of the MAS inhibitor AOAA (5 mM) is indicated. (**B**) Representative perforated-patch current clamp recording of spontaneous firing in wild-type (top) and MCU-KO (bottom) SNc neurons before and 15 min after bath application of AOAA (5 mM); scale bars, 10 mV, 2 s. (**C**) Quantification of the effect of 5 mM AOAA on firing on wild-type and MCU-KO SNc neurons (wild type: *n* = 8, *N* = 7; MCU-KO: *n* = 8, *N* = 5; *P* = 0.0195, one-tailed Wilcoxon matched-pairs signed rank test). (**D**) Representative 2PLSM ratio imaging time series showing the effect of bath application of AOAA (5 mM) followed by oligomycin (10 μM) and substitution of glucose with 2-DG on PercevalHR ratio in SNc dopaminergic neurons from wild-type (gray) and MCU-KO (black) neurons. (**E**) Quantification of the effect of AOAA (5 mM) on ATP/ADP ratio in wild-type and MCU-KO SNc neurons expressing PercevalHR (wild type: *n* = 5, *N* = 3; MCU-KO: *n* = 6, *N* = 4; *P* = 0.032, two-tailed Wilcoxon matched-pairs signed rank test). (**F**) Metabolites significantly changed in SNc tissue from MCU-KO mice versus wild-type mice (*N* = 5 for each group) identified by volcano plot, with fold change threshold (*x* axis) 2 and *t* test threshold (*y* axis) 0.1. Both fold changes and *P* values are log-transformed. (**G**) α-KG levels in wild-type and MCU-KO SNc tissue (*N* = 5 for each group. ^#^Statistical significance is based on (F). Box plots indicate first and third quartiles, thick center lines represent medians, and whiskers indicate the range. *P < 0.05.

Ca^2+^ release from the ER at MAMs leads to an elevation in Ca^2+^ concentration in the intermembrane space, in addition to a flux through the MCU into the matrix (*47*). The elevation in intermembrane Ca^2+^ can increase pyruvate availability and stimulate the malate-aspartate shuttle (MAS), which moves reducing equivalents derived from glycolysis into the mitochondrial matrix, where they can fuel the ETC (Fig. 7A).

If compensatory MAS activity accounts for the modest effects of MCU deletion, then its inhibition should have a more profound effect on dopaminergic neurons lacking the MCU than in controls. To test this hypothesis, aminooxyacetic acid (AOAA; 5 mM), which inhibits MAS by blocking aspartate aminotransferase (*48*), was used. In wild-type neurons, bath application of AOAA did not significantly change spike rate, suggesting that the MAS was not necessary for cytosolic ATP maintenance (Fig. 7, B and C). In contrast, MAS inhibition significantly slowed spiking in MCU-KO dopaminergic neurons—completely silencing some and driving them to a relatively depolarized membrane potential (Fig. 7, B and C). Consistent with this effect on spiking, MAS inhibition in MCU-KO neurons induced a more profound drop in cytosolic ATP/ADP ratio than in wild-type neurons (Fig. 7, D and E). These results suggest that MCU-mediated Ca^2+^ entry into the mitochondrial matrix and Ca^2+^-stimulated MAS activity work in tandem to support the ATP production necessary to support repetitive spiking in SNc dopaminergic neurons.

Although the MAS played a more substantial regulatory role in MCU-KO neurons, the expression of MAS-related genes was unchanged in MCU-KO neurons (fig. S9A). Another way in which MAS activity might be up-regulated is by increasing the availability of α-ketoglutarate (α-KG)—a key MAS substrate (*49*). Metabolomic analysis revealed a robust elevation in α-KG concentration in the SNc of MCU-KO mice (Fig. 7, F and G).

**Fig. 8.**
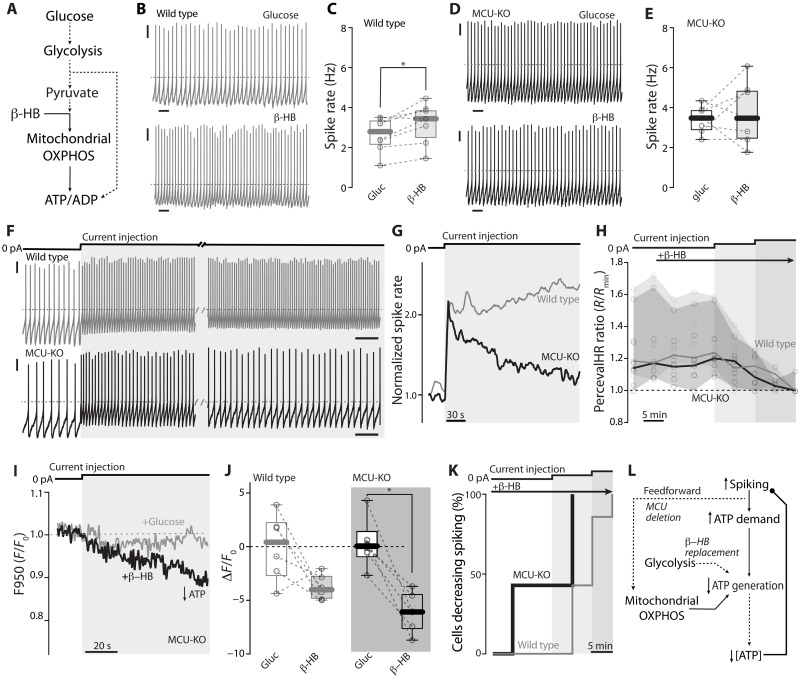
Bioenergetic deficiency in MCU-KO neurons relying on a mitochondrial-selective substrate. (**A**) Schematic illustrating the substitution of glucose with mitochondrial substrate β-HB (3.5 mM). (**B**) Representative perforated-patch recordings of wild-type neurons before and after (15 min) the substrate change. (**C**) Spike rate in wild-type neurons in glucose and β-HB aCSF (*n* = 8, *N* = 8; *P* = 0.0156, two-tailed Wilcoxon matched-pairs signed rank test). (**D**) Representative perforated-patch recordings of MCU-KO neurons before and after (15 min) the substrate change. (**E**) Spike rate in MCU-KO neurons in glucose and β-HB aCSF (*n* = 7, *N* = 7). (**F**) Representative perforated-patch recordings from wild-type (top, gray) and MCU-KO (bottom, black) neurons in β-HB aCSF at the onset and after 4 min of the depolarizing step. (**G**) Representative plots of normalized spike rate for wild-type (gray) and MCU-KO (black) neurons during the depolarizing step in β-HB aCSF. (**H**) PercevalHR ratio during the substrate change and depolarizing steps for wild-type (gray) and MCU-KO (black) SNc neurons; medians, range, and individual points are depicted (both *n* = 7, *N* = 7). (**I**) Representative PercevalHR F950 traces for MCU-KO neurons in glucose (gray) and β-HB aCSF (black) upon depolarization step. (**J**) Drop in PercevalHR F950 upon stimulation in glucose and β-HB aCSF for wild-type and MCU-KO neurons (both *n* = 6, *N* = 6; *P* = 0.0312, two-tailed Wilcoxon matched-pairs signed rank test). (**K**) Cumulative probability plots of cells spontaneously decreasing spike rate at different time points for wild-type (gray) and MCU-KO (black) neurons during the substrate change and depolarizing steps (both *n* = 7, *N* = 7; *P* = 0.009, Mantel-Cox test). (**L**) Schematic illustrating how MCU deletion combined with a mitochondria-selective substrate uncovers a bioenergetic deficit in SNc neurons. (B, D, and F) Scale bars, 10 mV, 1 s; dashed gray line, −40 mV. (F to K) Depolarizing steps, 50 pA. Box plots indicate first and third quartiles, thick center lines represent medians, and whiskers indicate the range. *P < 0.05.

To further assess the interplay of the MCU and MAS in regulating cytosolic ATP levels and spiking, glucose was replaced by β-hydroxybutyrate (β-HB) in the solution bathing neurons (3.5 mM). This switch disrupts glycolysis and the MAS but enables the TCA cycle to continue (Fig. 8A) (*50*). The basal spiking rate of MCU-KO dopaminergic neurons was not affected by β-HB substitution, while wild-type neurons displayed a small, but consistent, increase in rate (Fig. 8, B to E). However, when neurons were driven to spike above their basal rate, the impact of MCU deletion emerged. In perforated-patch recordings, intrasomatic current injection led to acceleration in spiking rate in both wild-type and MCU-KO neurons (Fig. 8F). However, unlike wild-type neurons, MCU-KO neurons were unable to sustain their elevated spiking rate (Fig. 8, F and G, and fig. S10A). Somewhat unexpectedly, the cytosolic ATP/ADP ratio measured every 5 min was very similar in wild-type and MCU-KO neurons throughout the period of current injection (Fig. 8H).

One interpretation of this result is that MCU-KO neurons engaged a feedback mechanism to slow spiking when ATP production failed to keep pace with demand. This type of mechanism would keep the cytosolic ATP/ADP ratio from dropping precipitously—much like what was seen when neurons were stimulated in the presence of isradipine (Fig. 5, B to F). If this was the case, then the ATP/ADP ratio should at least transiently decline following stimulation. To test this hypothesis, wild-type and MCU-KO SNc dopaminergic neurons were induced to express PercevalHR (as described above) and then recorded from using perforated-patch methods in ex vivo brain slices bathed in β-HB rather than glucose. To get better time resolution with PercevalHR, only fluorescence evoked by 950 nm illumination was measured to estimate the relative ATP concentration (*17*). As predicted, intrasomatic current injection produced a rapid decline in cytosolic ATP concentration in MCU-KO neurons relying upon β-HB and the TCA cycle alone (Fig. 8, I and J). There also was a trend for cytosolic ATP levels to decline with stimulation in wild-type neurons, but this trend did not reach statistical significance (Fig. 8J and fig. S10B). As shown in a survival plot of spiking rate (Fig. 8K), MCU-KO neurons manifested a greater sensitivity to β-HB substitution than did wild-type neurons. Again, these results are consistent with the hypothesis that the MCU is a key element in the feedforward signaling mechanism helping to match anticipated ATP demand to ATP generation, which is critical to sustained spiking (Fig. 8L).

## DISCUSSION

The studies presented demonstrate that plasma membrane Ca_v_1 Ca^2+^ channels trigger an intracellular Ca^2+^ signaling cascade that engages ER RYRs to stimulate mitochondrial ATP synthesis to help meet the bioenergetic demand accompanying regenerative activity in SNc dopaminergic neurons. RYR-dependent Ca^2+^ release stimulated mitochondrial OXPHOS by increasing Ca^2+^ flux into the matrix through the MCU, as well as by elevating the activity of the MAS. In so doing, this tandem feedforward pathway enabled SNc dopaminergic neurons to sustain cytosolic ATP levels and regenerative activity (spiking) in response to prolonged excitatory drive. Given the importance of basal ganglia dopamine release to sequential, goal-directed movement, this regulatory network is very likely to have been of survival value and, hence, selected for by evolution. However, this design may also put SNc dopaminergic neurons at risk for mitochondrial dysfunction with aging, environmental toxins, and genetic mutations associated with PD.

### A role for Ca_v_1.3 channels in bioenergetic control

For roughly 30 years, the prevailing view has been that Ca^2+^ flux through Ca_v_1 channels provides a subthreshold, depolarizing current that helps create the negative slope conductance region necessary for autonomous pacemaking in SNc dopaminergic neuron (*41*, *42*, *51*). The evidence for this conclusion was based on the ability of dihydropyridines (DHPs), which are NAMs of Ca_v_1 channels to stop pacemaking and to suppress membrane potential oscillations in the presence of the Na_v_1 Na^+^ channel blocker tetrodotoxin (*52*, *53*). Because SNc dopaminergic neurons coexpress both high voltage–activated Ca_v_1.2 channels and low voltage–activated Ca_v_1.3 channels (*54*, *55*), this subthreshold electrogenic role has been ascribed specifically to Ca_v_1.3 channels (*42*). However, when intracellular ATP concentrations are clamped by whole-cell dialysis, inhibition of both Ca_v_1.2 and Ca_v_1.3 channels with channel-specific DHP concentrations does not slow basal pacemaking despite blunting intracellular Ca^2+^ oscillations (*19*).

While our results do not exclude the possibility that inward current carried by Ca_v_1 channels contributes to electrogenesis at spiking rates above baseline, they point clearly to an alternative function in bioenergetic control. In all neurons, regenerative activity dissipates Na^+^, K^+^, and Ca^2+^ gradients that must be restored by ATP-dependent pumps and transporters (*1*, *56*). Computational studies suggest that even modest deficits in mitochondrial ATP production can compromise the ability of neurons to maintain these ionic gradients when neurons are driven to spike repetitively (*3*). Our results show that the capacity of mitochondria to produce ATP is continuously tuned to match demand by the activity-dependent opening of Ca_v_1 channels. When the coupling between Ca_v_1 channels and mitochondria was broken, either pharmacologically or genetically, SNc dopaminergic neurons were not able to sustain their cytosolic ATP/ADP ratio when driven to spike at rates above baseline. This bioenergetic deficit increased the irregularity of spiking and, as it worsened, caused cessation of spiking and membrane depolarization—as predicted by a failure to maintain adequate Na^+^/K^+^ ATPase activity (*3*). This phenomenon resembles the depolarization block observed in dopaminergic neurons treated with antipsychotics and overexcitation (*57*, *58*). The inference that this deficit had bioenergetic, as opposed to electrogenic, origins was also supported by the ability of elevated concentrations of extracellular glucose, which increases glycolytic ATP production, to reverse the effects of Ca_v_1 channel inhibition on spiking rate and regularity.

Another set of observations support the inference that Ca_v_1 channels control mitochondrial OXPHOS in SNc dopaminergic neurons. Deletion of the gene coding for the Ndufs2 subunit of mitochondrial complex I (MCI) results in a profound disruption in OXPHOS and an up-regulation in glycolysis (*39*). This perturbation, which makes mitochondrial OXPHOS unresponsive to Ca^2+^ signaling, results in down-regulation of Ca_v_1 channel expression, shortening of action potentials, and a profound reduction in spike-linked cytosolic Ca^2+^ transients in SNc dopaminergic neurons (*39*)—pointing to a pivotal role for Ca_v_1 channels in metabolic regulation. Going forward, it will be important to define the relative roles of Ca_v_1.2 and Ca_v_1.3 Ca^2+^ channels in this bioenergetic control mechanism.

### A key role for RYRs in metabolic coupling

The coupling of Ca_v_1 channels to regulation of mitochondrial OXPHOS in SNc dopaminergic neurons was mediated by a mechanism similar to that described in muscle cells (*59*, *60*). In muscle cells, the close physical proximity between RYRs and the cytoplasmic domain of Ca_v_1 channels allows Ca^2+^ flux through them to efficiently couple spike activity to RYR-dependent CICR. This coupling was evident in SNc dopaminergic neurons too, as inhibition of RYR opening cut the cytoplasmic Ca^2+^ transient induced by Ca_v_1 channel opening nearly in half. While in principle this linkage creates a way in which Ca_v_1 channels might regulate ER function, spike-dependent RYR opening did not measurably alter luminal Ca^2+^ concentration assessed with the CEPIA probe. It is possible that there were local changes in ER Ca^2+^ content that were not detected, but it is also likely that the ER Ca^2+^ flux was not great enough to noticeably reduce the heavily buffered ER Ca^2+^ concentration (*61*).

CICR can generate a propagating Ca^2+^ wave in the ER, linking spatially separated regions of the cell (*62*). In SNc dopaminergic neurons, RYR-dependent Ca^2+^ release functionally coupled plasma membrane Ca_v_1 channels to mitochondria that are commonly docked to the ER at specialized junctions or MAMs (*28*, *63*). The relationship between spike rate and somatic mitochondrial matrix Ca^2+^ measured with mito-GCaMP6 was roughly linear. In dendrites, this relationship was steeper at low discharge rates and then plateaued. Ca^2+^ entry into the mitochondrial matrix through the MCU is an important positive modulator of the TCA cycle and OXPHOS (*37*, *43*). The subcellular differences may reflect an adjustment in mitochondrial coupling to match regional variation in the bioenergetic cost of a spike; as the ratio of membrane surface area to cytoplasmic volume is higher in dendrites, the cost of actively propagated spikes per mitochondrial unit should be higher if mitochondrial density is uniform (*64*). The subcellular differences in mitochondrial Ca^2+^ loading (Figs. 2G and 3E) parallel estimates of mitochondrial matrix oxidant stress in these neurons (*15*, *65*), underscoring the link between mitochondrial Ca^2+^, OXPHOS, and oxidant stress. This relationship was not seen in neighboring VTA dopaminergic neurons (Fig. 2F) (*15*, *65*). Although our focus was on regenerative activity, this difference might also reflect other activity-dependent dendritic bioenergetic demands, such as transmitter release and sequestering dopamine (*66*).

### A tandem Ca^2+^ couple to mitochondrial OXPHOS

As important as MCU-mediated Ca^2+^ flux into the mitochondrial matrix appears to be, knocking the MCU out of SNc dopaminergic neurons had very modest effects on cytosolic ATP/ADP ratio and the ability to sustain repetitive spiking. Similarly, previous studies probing the bioenergetic impact of MCU deletion on nonneuronal cells have found clear effects of MCU deletion only in metabolically demanding situations, like those induced by strenuous exercise (*18*, *67*–*69*). In large part, this resilience is attributable to the engagement of the MAS. ER Ca^2+^ release stimulates the MAS component Aralar in the intermembrane space and increases shuttling of reducing equivalents generated through glycolysis to mitochondria (*70*, *71*). Although there were no significant changes in the expression of MAS-related genes following MCU deletion, when matrix Ca^2+^ uptake is blunted, α-KG—a MAS substrate—–rises, boosting the shuttling of reducing equivalents for the ETC (*49*, *72*). Consistent with an inferred compensation, in MCU-KO neurons, α-KG was elevated, and disrupting MAS function with AOAA led to a drop in the cytosolic ATP/ADP ratio, membrane depolarization, and cessation of spiking.

This tandem, Ca^2+^-dependent regulation of mitochondrial OXPHOS endows SNc dopaminergic neurons with a flexible and robust feedforward control system that couples spiking, which is an excellent predictor of bioenergetic needs, and ATP production. SNc dopaminergic neurons were resistant to perturbations in either one of these mitochondrial control networks, despite the clear-cut reliance upon OXPHOS for ATP production. This dual control should also help SNc dopaminergic neurons maintain adequate ATP production in the face of changing energy sources. For example, metabolism of alternative energy substrates, including ketone bodies and lactate transferred from astrocytes (*50*, *73*), appeared to be effectively managed by Ca^2+^ stimulation of the TCA cycle, as glucose substitution with β-HB only affected spiking in the absence of the MCU. This flexibility is likely crucial in natural environments, where food varies in composition and availability. It is likely that in this situation (in contrast to the laboratory environment), the ability of the MAS to compensate for MCU deletion would be limited, as the MAS relies upon reducing equivalents generated by glycolysis.

Although this feedforward control system is very likely to be a common feature of excitable cells, its “gain” appears to be relatively high in SNc dopaminergic neurons and in other neurons with a similar phenotype. SNc dopaminergic neurons not only robustly express Ca_v_1 channels that open before and during spiking (*41*) but also have low intrinsic Ca^2+^ buffering (*74*), high expression of MICU3 [a high-affinity Ca^2+^ sensor of the MCU complex (*75*, *76*)], and high expression of the low-affinity isoform of SERCA (SERCA3) (fig. S8) (*77*). Noradrenergic neurons in the locus coeruleus (LC), which have many of the same physiological and anatomical traits (*78*, *79*), also respond to DHP inhibition of Ca_v_1 channels with a similar disruption of spiking when recorded with methods that do not perturb their metabolism (*79*). While our functional assays used spiking to assess the extent to which neurons were bioenergetically compromised by disrupting feedforward control, it is highly likely that a wide array of anabolic and catabolic processes was compromised as well (*80*, *81*). Furthermore, the bioenergetic demands of these cellular processes, both at baseline and during periods of high activity, may be particularly large in neurons that have extensive axonal arbors, such as SNc dopaminergic and LC noradrenergic neurons, creating a condition in which the “spare capacity” of OXPHOS is very limited, underscoring the functional importance of a dynamic OXPHOS control mechanism (*14*, *82*, *83*).

### Metabolic control and antagonistic pleiotropy

As important as feedforward control of mitochondrial OXPHOS is for meeting the bioenergetic needs of SNc dopaminergic neurons during stimulated activity, there are negative consequences as well that may manifest themselves with aging (*14*, *84*). The cardinal motor symptoms of PD are caused by the aging-dependent degeneration of SNc dopaminergic neurons. A hallmark of the SNc in PD patients is the accumulation of mitochondrial DNA deletions and other signs of sustained oxidant stress, such as the loss of MCI function (*85*–*88*). Loss of MCI function is sufficient to trigger a progressive, human-like form of levodopa-responsive Parkinsonism in mice (*39*). Ca_v_1 channel–mediated feedforward control of mitochondrial OXPHOS in SNc dopaminergic neurons may be primary driver of mitochondrial damage in PD. While the generation of reactive oxygen species and oxidant stress is an inevitable consequence of OXPHOS, it is exacerbated by stimulation of the ETC in the absence of dissipative MCV activity (*89*). That is, when the ETC is stimulated in the absence of ATP demand, oxidant stress rises. Feedforward stimulation of the ETC in anticipation of bioenergetic demand is likely to increase this mismatch. Ca_v_1 channel signaling increases basal mitochondrial oxidant stress in SNc dopaminergic neurons (*15*, *25*). In animal models, systemic DHP administration, which blunts feedforward signaling, substantially diminishes mitochondrial damage and turnover (*25*). Moreover, epidemiological studies have consistently found that human use of DHPs diminishes the risk of developing PD, and recent reanalyses of clinical trial data with the DHP isradipine are consistent with a protective, disease-modifying effect (*90*–*96*). Although partial inhibition of Ca_v_1 Ca^2+^ channels and feedforward control of mitochondrial OXPHOS may compromise the ability of SNc dopaminergic neurons to mobilize basal ganglia circuitry and promote rapid, goal-directed movement in stressful circumstances, this trade-off would seem to be an acceptable one for most of society, particularly with aging. In building out the mechanistic connection between Ca_v_1 channels, mitochondria, and PD risk, our studies have identified other potential disease-modifying targets.

## MATERIALS AND METHODS

### Animals

C57/BL6, B6.SJL-Slc6a3tm1.1(5 cre)Bkmn/J (DAT-Cre) (*97*), and MCU^fl/fl^ (*18*) mice were bred in-house and used with the approval by Northwestern University Animal Care and Use Committee and in accordance with the National Institutes of Health (NIH) *Guide for the Care and Use of Laboratory Animals*. Mice were group-housed with food and water ad libitum on a 12-hour light/dark cycle with temperatures of 65° to 75°F and 40 to 60% humidity. MCU^fl/fl^ mice were bred with DAT-Cre mice to obtain DAT-Cre^+/−^ MCU^fl/fl^ and DAT-Cre^−/−^ MCU^fl/fl^ mice (“MCU-KO” and “wild type”). For RiboTag experiments, DAT-Cre^+/−^ mice, generated breeding DAT-Cre^+/+^ and C57/BL6 mice, were used as controls for DAT-Cre^+/−^ MCU^fl/fl^ mice. C57/BL6 mice were sacrificed for experiments between postnatal day 26 (p26) and p40, while MCU-KO mice (and corresponding controls) were sacrificed for experiments between p40 and p50, and at 1 year for behavioral experiments.

### Virus generation

PCR-amplified sequences for GCaMP6s (*16*), 2mt-GCaMP6m (*29*), G-CEPIA1er (*27*), PercevalHR (*17*), and mito-roGFP (*98*) were subcloned into Eco RI and Sal I restriction sites of the pFB-TH-SV40 vector and packaged into recombinant AAVs, serotype 9, titers ~2 × 10^13^ vp (viral particles)/ml (Virovek).

### Stereotaxic surgery

An isoflurane precision vaporizer (Smiths Medical PM) was used to anesthetize mice. Mice were then placed on a stereotaxic frame (David Kopf Instruments), with a Cunningham adaptor (Harvard Apparatus) to maintain anesthesia delivery during surgery. The skull was exposed, and a small hole was drilled at the desired injection site. The following stereotaxic coordinates were used: SNc, 3.1 and 1.3 mm posterior and lateral to bregma, at a depth of 4.5 mm from dura; dorsolateral striatum, 0.34 and 2.5 mm anterior and lateral to bregma, at a depth of 3 mm from dura. The Allen Mouse Brain Atlas, online version 1, 2008 (https://atlas.brain-map.org/) was used as a reference for the coordinates and generating diagrams.

For each mouse, the distance between bregma and lambda was calculated and used to adjust the coordinates. For AAV injections, ~350 nl of viral vector was delivered using a glass micropipette (Drummond Scientific) pulled with a P-97 glass puller (Sutter Instruments). Surgery for imaging experiments was performed unilaterally, and surgeries for RiboTag tissue collection were executed bilaterally. Experiments were performed after at least 10 postoperative days; tissue collection for RiboTag was performed 4 weeks after injection.

### Ex vivo slice preparation

Mice were terminally anesthetized with a mixture of ketamine (50 mg/kg) and xylazine (4.5 mg/kg) and transcardially perfused with ice-cold modified artificial cerebrospinal fluid (aCSF) (“slicing solution,” containing 49.14 mM NaCl, 2.5 mM KCl, 1.43 mM NaH_2_PO_4_, 25 mM NaHCO_3_, 25 mM glucose, 99.32 mM sucrose, 10 mM MgCl_2_, and 0.5 mM CaCl_2_); after decapitation, the brain was removed and sectioned in 220-μm-thick coronal slices using a vibratome (VT1200S, Leica Microsystems) in the same slicing solution.

Slices were transferred into a holding chamber containing recording aCSF (“physiological glucose aCSF,” containing 135.75 mM NaCl, 2.5 mM KCl, 1.25 mM NaH_2_PO_4_, 25 mM NaHCO_3_, 2 mM CaCl_2_, 1 mM MgCl_2_, and 3.5 mM glucose). Slices were allowed to recover for 30 min at 34°C and then kept at room temperature for at least 15 min before starting the experiments.

For specific experiments, aCSF was modified by replacing glucose (3.5 mM) with the same concentration of alternative substrates: 2-DG (3.5 mM, Sigma-Aldrich) and β-HB (3.5 mM, Sigma-Aldrich). High-glucose aCSF contained 125 mM NaCl, 2.5 mM KCl, 1.25 mM NaH_2_PO_4_, 25 mM NaHCO_3_, 2 mM CaCl_2_, 1 mM MgCl_2_, and 25 mM glucose. When comparing the effect of acute transition from low- to high-glucose aCSF, a modified low-glucose aCSF was adopted where glucose was partially substituted with sucrose to preserve osmolarity without changing the NaCl concentration: 125 mM NaCl, 2.5 mM KCl, 1.25 mM NaH_2_PO_4_, 25 mM NaHCO_3_, 2 mM CaCl_2_, 1 mM MgCl_2_, 3.5 mM glucose, and 21.5 mM sucrose.

All solutions were pH 7.4 and ~310 mOsm, and continually bubbled with 95% O_2_ and 5% CO_2_. For isradipine preincubation experiments, after recovery, slices were transferred to a holding chamber containing physiological glucose aCSF with 0.5 μM isradipine for at least 1 hour before starting the experiments.

### 2PLSM optical workstation description

The laser scanning optical workstation embodies an Ultima dual-excitation-channel scan head (Bruker Nano Fluorescence Microscopy Unit). The foundation of the system is the Olympus BX-51WIF upright microscope with an LUMPFL 60×/1.0 NA (numerical aperture) water-dipping objective lens. The automation of the XY stage motion, lens focus, and manipulator XYZ movement was provided by FM-380 shifting stage, axial focus module for Olympus scopes, and manipulators (Luigs & Neumann). Cell visualization and patching were made possible by a variable magnification changer, calibrated to 2× (100-μm field of view) as defined by the LSM bright-field transmission image, supporting a 1-megapixel USB3.0 complementary metal-oxide semiconductor (CMOS) camera (DCC3240M, Thor Labs) with ~30% quantum efficiency around 770 nm. Olympus NIR-1 band-pass filter, 770 nm/100 nm, and μManager (*99*) software were used with the patch camera. The electrical signals were sent and collected with a 700B patch clamp amplifier, and MultiClamp Commander software with computer input and output signals was controlled by Prairie View 5.3-5.5 using a National Instruments PCI-6713 output card and PCI-6052e input card.

The 2P excitation (2PE) imaging source was a Chameleon Ultra1 series tunable wavelength (690 to 1040 mm, 80 MHz, ~250 fs at sample) Ti: sapphire laser system (Coherent Laser Group); the excitation wavelength was selected on the basis of the probe being imaged (see below). Each imaging laser output is shared (equal power to both sides) between two optical workstations on a single anti-vibration table (TMC). Workstation laser power attenuation was achieved with two Pockels’ cell electro-optic modulators (models M350-80-02-BK and M350-50-02-BK, Con Optics) controlled by Prairie View 5.3-5.5 software. The two modulators were aligned in series to provide enhanced modulation range for fine control of the excitation dose (0.1% steps over 5 decades), to limit the sample maximum power, and to serve as a rapid shutter during line scan or time series acquisitions.

The 2PE generated fluorescence emission was collected by non–de-scanned photomultiplier tubes (PMTs). Green channel (490 to 560 nm) signals were detected by a Hamamatsu H7422P-40 select GaAsP PMT. Red channel (580 to 630 nm) signals were detected by a Hamamatsu R3982 side on PMT. Dodt-tube–based transmission detector with Hamamatsu R3982 side on PMT (Bruker Nano Fluorescence) allowed cell visualization during laser scanning. Scanning signals were sent and received by the National Instruments PCI-6110 analog-to-digital converter card in the system computer (Bruker Nano Fluorescence). All XY images were collected with a pixel size of 0.195 μm, a pixel dwell time of 12 μs, and a frame rate of 3 to 4 fps (frames per second).

### Ex vivo Ca^2+^ 2PLSM measurements

Slices were transferred to a recording chamber and continuously perfused with aCSF at 32° to 33°C (unless otherwise specified). A laser wavelength of 920 nm was used for GCaMP6s, mito-GCaMP6, and G-CEPIA1er experiments. Alternatively, brightness over time (BOT) continuous measurements were collected during experiments with acute stimulation.

To calibrate the dynamic range of GCaMP6s, mito-GCaMP6, and G-CEPIA1er probes in the experiments, slices were perfused with Ca^2+^-free aCSF (CaCl_2_ was substituted with MgCl_2_, for a total of 3 mM MgCl_2_) + 0.5 mM EGTA and 1 μM ionomycin to obtain *F*_min_, followed by aCSF (with total 3 mM CaCl_2_) + 1 μM ionomycin to obtain *F*_max_.

Time series and BOT data were analyzed offline, and fluorescence measurements in multiple regions of interest (ROIs) were evaluated, with a separate background ROI value subtracted. Measurements are presented as *F* − *F*_min_, where *F*_min_ is the minimum fluorescence obtained in Ca^2+^-free aCSF + ionomycin. Semiquantitative estimations of Ca^2+^ levels were calculated as the percentage of the dynamic range of the probe (% of MAX). The mitoGCaMP6 experiments comparing somatic and dendritic mitochondria were performed at room temperature. For mito-GCaMP6 experiments combined with perforated-patch electrophysiological recordings (see below), continuous BOT measurements were performed and the minimum fluorescence was obtained by silencing the cell by injecting hyperpolarizing currents.

### Ex vivo redox 2PLSM measurements

Mito-roGFP experiments were performed using an excitation wavelength of 920 nm, as previously described (*15*). Briefly, time series images were acquired with 60 frames obtained over ~20 s. The dynamic range of the probe was determined by bath applying 2 mM dithiothreitol, a reducing agent, followed by 200 μM aldrithiol, an oxidizing agent, to determine the fluorescence intensities of the probe at minimal and maximal oxidation for each cell. Time series were analyzed offline, multiple ROIs were measured, the background ROI value was subtracted, and then the relative oxidation was calculated.

### Ex vivo ATP/ADP 2PLSM measurements

For the excitation ratio probe PercevalHR, ATP/ADP ratio measurements of two excitation wavelengths, 950 nm for ATP and 820 nm for ADP, were used in rapid succession for each acquisition time point and two time series of five frames (1.25 to 1.7 s long) were acquired for each wavelength. Time series were analyzed offline, a cytosolic and a background ROIs were measured, the background signal was subtracted, and the ATP/ADP ratio (950:820) was calculated for each ratio pair time point.

The contribution of mitochondria to the bioenergetic status of each cell (OXPHOS index) was estimated comparing the drop in the PercevalHR ATP/ADP ratio induced by bath application of 10 μM oligomycin (Sigma-Aldrich) and the minimum ratio obtained in a modified aCSF where glucose was substituted with the nonhydrolyzable 2-DG (see above) plus 10 μM oligomycin. For experiments with other pharmacological manipulations, the ATP/ADP ratio is presented as *R* − *R*_min_, where *R* is the ratio measured at the time point of interest and *R*_min_ is the minimum ratio obtained in 2-DG aCSF + 10 μM oligomycin. For PercevalHR experiments combined with perforated-patch electrophysiological recordings (see below) where the acute effect of current injection was analyzed, only the ATP-sensitive wavelength (950 nm) was used as images were continuously collected as a BOT series.

### Electrophysiology and Fura-2 2PLSM Ca^2+^ imaging

Slices were transferred in a recording chamber on an Olympus BX51 upright microscope and perfused with oxygenated aCSF as described above. Patch pipettes were pulled from thick-walled borosilicate glass on a Sutter P-1000 puller; pipette resistance was 2.5 to 5 megohms. Electrophysiological recordings were obtained using a MultiClamp 700B amplifier. For most recordings, electrophysiological signals were filtered at 1 to 4 kHz and digitized at 10 kHz. The liquid junction potential in the recording aCSF was 9.6 mV and corrected for.

For experiments performed in perforated-patch configuration, pipettes were front-filled using a potassium-based internal solution containing 126 mM KMeSO_4_, 14 mM KCl, 10 mM Hepes, 1 mM EGTA, 0.5 mM CaCl_2_, and 3 mM MgCl_2_; pH was adjusted to 7.25 to 7.3 with KOH, osmolarity 280 to 300 mOsm; Alexa Fluor 594 hydrazide (25 μM; Thermo Fisher Scientific) was added to the internal solution. The pipette was then back-filled with the same solution containing gramicidin D (20 μg/ml; Sigma-Aldrich), as in (*100*). After obtaining a gigaseal, cells were left to perforate until the spike height approached 0 mV before data collection began. Electrophysiological recordings were normally coupled with 2PLSM experiments, with the settings indicated above for the different genetically encoded probes. In addition, the Alexa Fluor 594 fluorescence was monitored using 820 nm as excitation wavelength. Rapid jumps in the observed voltage values or the appearance of Alexa Fluor 594 signal inside the patched cell indicated that break-in had occurred, after which the recording was stopped.

For Fura-2 imaging, conventional tight-seal whole-cell patch-clamp recordings were made from SNc dopaminergic neurons. The patch electrode solution contained 135 mM KMeSO_4_, 5 mM KCl, 5 mM Hepes, 10 mM Na_2_PCr (phosphocreatine disodium), 2 mM ATP-Mg, and 0.5 mM guanosine triphosphate (GTP)–Na, pH ~ 7.3 and osmolarity 290 to 300 mOsm, with addition of Fura-2 (100 μM).

A 780-nm laser wavelength was used to perform 2PLSM Fura-2 Ca^2+^ imaging using line scan acquisitions with 0.195-μm pixels and 12-μs dwell time, and [Ca^2+^] was estimated using the following equation: [Ca^2+^] = *K*_d_ [1 − *f*(*t*)/*f*_max_]/[*f*(*t*)/*f*_max_ − 1/*R*_f_], as previously described (*25*). A *K*_d_ of 200 nM and *R*_f_ of 20 were assumed and used.

### Immunofluorescence and confocal imaging

Fixed tissue was prepared by transcardially perfusing terminally anesthetized mice (see above) with phosphate-buffered saline (PBS; Sigma-Aldrich) immediately followed by 4% paraformaldehyde (PFA; diluted in PBS from a 16% stock solution; Electron Microscopy Sciences). The brain was then removed and transferred into PFA solution for 1 to 4 hours before being thoroughly rinsed and stored in PBS at 4°C. Fixed brains were then sectioned into 50- to 60-μm-thick coronal slices on a Leica VT1200S vibratome and collected in PBS.

Sections were bathed in a permeabilization solution (0.5% Triton X-100 in PBS) for 15 min, quickly washed with PBS, and then bathed in blocking solution [10% normal goat serum (NGS) and 0.25% Triton X-100 in PBS] for 30 min before primary antibodies, diluted at the appropriate concentration in the same blocking solution, were applied overnight at 4°C.

The following primary antibodies were used: anti-TH (mouse, Immunostar 22941, 1:1000), anti-GFP (rabbit, Life Technologies, A-11122, 1:1000), anti-GFP (mouse, Millipore, MAB3580, 1:1000), anti-COXIV (rabbit, Proteintech, 11242-1-AP, 1:200), and anti-CRT (rabbit, Pierce, PA3-900, 1:200).

Subsequently, sections were washed three times in PBS and then incubated in secondary antibodies with the desired fluorophores (Alexa Fluor, Life Technologies) diluted (1:500) in PBS with 10% NGS for 1 hour at room temperature. After three washes in PBS, sections were transferred on microscopy slides (VWR) and allowed to dry and mounted with ProLong Diamond (Thermo Fisher Scientific) and #1.5 glass coverslips (VWR).

Mounted sections were stored at 4°C until imaged with an Olympus FV10i confocal laser scanning microscope, using 10×/0.4 (air) or 60×/1.35 (oil) objective. FIJI (NIH) was used to adjust images for brightness, contrast, and pseudo-coloring.

### Fluorogold and transmission electron microscopy

Mice were stereotactically injected into the dorsolateral striatum with 300 nl of 1% Fluorogold (Santa Cruz Biotechnology) dissolved in 0.9% NaCl saline as described above. Six days after surgery, mice were transcardially perfused with PBS, followed by a fixative containing 2% PFA, and 1.25% glutaraldehyde in 0.1 M cacodylate buffer (pH 7.3; Electron Microscopy Sciences). Brains were extracted and incubated overnight with fixative solution and rinsed thoroughly with PBS, and 400-μm midbrain slices were obtained with a Leica VT1200S vibratome.

Upon visual inspection of the slices, a 1 mm by 1 mm square containing the SNc region with the best Fluorogold retrograde labeling was dissected for each brain and transferred back into the fixative solution. Dissected areas were then postfixed in buffered 2% OsO_4_, rinsed and stained in 3% uranyl acetate, dehydrated in ascending ethanol, transitioned with propylene oxide, and embedded in Embed 812 (Electron Microscopy Sciences). A Leica Ultracut UC6 ultramicrotome was then used to section samples. Sections (1 μm thick) were stained with toluidine blue O, and 70-nm sections were collected on 200-mesh copper grids and stained with uranyl acetate and Reynolds lead citrate.

Samples were analyzed with a Tecnai Spirit G2 (FEI Company) transmission electron microscope operated at 80 kHz. SNc dopaminergic neurons were identified cytologically by a ribosome-rich cytoplasm, stacks of ER cisternae (*101*), and the presence of structures indicative of Fluorogold uptake (*102*). Transmission electron microscopy sample preparation and observation were carried out at the Center for Advanced Microscopy at Northwestern University.

### RiboTag profiling

AAVs for expression of RiboTag under a cre-dependent promoter (AAV9-EF1a-DIO-Rpl22l1-Myc-DDK-2A-tdTomato-WPRE, titers 2.24 × 10^13^ viral genomes/ml) were stereotaxically injected into the SNc in DAT-Cre^+/−^ and MCU-KO mice, as described above. Four weeks after injection, mice were sacrificed and the SNc tissue expressing RiboTag was dissected and frozen at −80°C. RiboTag immunoprecipitation was carried out as previously described (*103*). Briefly, tissue was homogenized in cold homogenization buffer [50 mM tris (pH 7.4), 100 mM KCl, 10 mM MgCl_2_, 1 mM dithiothreitol, cycloheximide (100 μg/ml), protease inhibitors, recombinant ribonuclease (RNase) inhibitors, and 1% NP-40]. Homogenates were centrifuged at 10,000*g* for 10 min, and the supernatant was collected and precleared with protein G magnetic beads (Thermo Fisher Scientific) for 1 hour at 4°C, under constant rotation. Immunoprecipitations were carried out with anti-Flag magnetic beads (Sigma-Aldrich) at 4°C overnight with constant rotation, followed by four washes in high-salt buffer [50 mM tris (pH 7.4), 350 mM KCl, 10 mM MgCl_2_, 1% NP-40, 1 mM dithiothreitol, and cycloheximide (100 μg/ml)]. RNA was extracted using RNeasy Micro RNA extraction kit (QIAGEN) according to the manufacturer’s instructions.

### Quantitative real-time PCR

RNA was extracted from the dissected SNc tissue using RNeasy mini kit (QIAGEN). cDNA was synthetized by using the SuperScript IV VILO Master Mix (Applied Biosystems) and preamplified for 10 cycles using TaqMan PreAmp Master Mix and pool of TaqMan Gene Expression Assays (Applied Biosystems). The resulting product was diluted and then used for PCR with the corresponding TaqMan Gene Expression Assay and TaqMan Fast Advanced Master Mix. Data were normalized to Hprt by the comparative CT (2-DDCT) method.

The following TaqMan probes were used for PCR amplification of *hprt* (Mm01318746_g1) and *Mcu* (custom made; up, ACATACCACGTACGGCCAC; low, ATGCTGCTCAATGCACAGT; probe, ACGCTGAACGACGTGAAGACCC) genes.

Experimental Ct values were normalized to hprt values using the following formula: ΔCt = Ct (*Mcu*) − Ct (*hprt*). The final expression levels were shown as ΔCt values.

### RNA sequencing

were trimmed to remove Illumina adapters from the 3′ ends using cutadapt (*104*). Trimmed reads were aligned to the *Mus musculus* genome (mm10) using STAR (*105*). Read counts for each gene were calculated using htseq-count (*106*) in conjunction with a gene annotation file for mm10 obtained from Ensembl (http://useast.ensembl.org/index.html). Normalization and differential expression were calculated using DESeq2, which uses the Wald test (*107*). The cutoff for determining significantly differentially expressed genes was a false discovery rate (FDR)–adjusted *P* value less than 0.05 using the Benjamini-Hochberg method. RNA-seq analysis was performed at the RNASeq Core at Northwestern University.

### Metabolomics

SNc was dissected and snap-frozen on dry ice and stored at −80°C until processed. Acetonitrile/water (80:20) equilibrated to −20°C was pipetted into each sample tube in a volume of 20 μl/mg of tissue (range, 2.4 to 6.1 mg). Tissue was mechanically dissociated by pipetting up and down. Samples were frozen on dry ice and thawed six times, each time followed by pipetting up and down, and then stored at −80°C overnight. Once thawed, samples were vortexed for 1 min and then centrifuged for 30 min at 4°C at 14,000*g*. The supernatant was then transferred to high-performance liquid chromatography (HPLC) tubes and stored at −80°C. Subsequently, samples were analyzed by ultrahigh-performance liquid chromatography and high-resolution mass spectrometry and tandem mass spectrometry, as described previously (*108*). Data acquisition and analysis were carried out by Xcalibur 4.1 software and Tracefinder 4.1 software, respectively (both from Thermo Fisher Scientific). The peak area for each detected metabolite was normalized by the total ion current, which was determined by integration of all the recorded peaks within the acquisition window. Metabolomics services were performed by the Metabolomics Core Facility at Robert H. Lurie Comprehensive Cancer Center of Northwestern University.

### Behavioral tests

Mice were habituated to the dimly lit and noise-controlled room for 60 min before the start of each test.

#### Open field test

Mice were placed in the open field box (56 cm by 56 cm), and locomotor activity was monitored over 5 min using LimeLight software (ACTIMETRICS). Total distance traveled, number of pauses, and speed were measured.

#### Adhesive removal test

The test was performed by placing the mice in a 500-ml beaker. Mice were allowed to habituate to the beaker for 30 min on the day before the test. The following day, two adhesive tape strips were applied with equal pressure on each animal’s forepaw. The total time required to remove the strips from both paws was measured starting from the moment when the mouse first felt the tape (i.e., the mouse brought its paws to its mouth), for a maximum time of 300 s (*109*).

#### Maximum speed evaluation

Mice were placed on a motorized treadmill. After a brief acclimation time, the treadmill was started (5 cm/s) and the belt speed was progressively increased. The maximum speed at which the mouse could sustain walking without stopping and being pulled to the back of the treadmill was recorded.

### Analysis and statistics

Images were analyzed offline using Fiji [NIH; (*110*)]. Traces were subsequently analyzed with Excel (Microsoft), GraphPad Prism 8 (GraphPad Software), or Igor Pro 5-8 (WaveMetrics).

Electrophysiology data were generally analyzed using Python with custom written analysis scripts as described in (*100*). The code is available at https://github.com/surmeierlab/EZT2022SA.

For Fura-2 Ca^2+^ imaging combined with electrophysiological recording, Clampfit 10.3 (Molecular Devices) and Igor Pro 5-8 (WaveMetrics) were used for analysis. Data are summarized using box plots showing median values, first and third quartiles, and range, unless otherwise specified.

Sample *n* represents the number of neurons collected from brain slices from *N* animals. Statistical analysis was performed with GraphPad Prism 8 (GraphPad Software), except for metabolomic data. Nonparametric tests (Mann-Whitney *U* test of significance or Wilcoxon signed rank test for nonpaired or paired design experiments, respectively) were used, unless otherwise stated. Two-tailed tests were used unless the working hypothesis predicted a clear directionality to the change in outcome measure, in which case one-tailed tests were adopted. Normality was assessed using the Shapiro-Wilk test. Comparison of survival curves was performed with the log-rank (Mantel-Cox) test. The Benjamini-Hochberg method was used to correct for multiple comparisons for RNA-seq data, reporting the FDR-adjusted *P* value. Probability threshold for statistical significance was *P* < 0.05. For metabolomic data, statistical analysis was performed using MetaboAnalyst 5.0 (*111*) using *t* test and volcano plot generation.

## Notes

View/request a protocol for this paper from *Bio-protocol*.
