# Supplementary material for: Ca2+ channels couple spiking to mitochondrial metabolism in substantia nigra dopaminergic neurons
Source: Sci Adv. 2022 Sep 30;8(39):eabp8701. doi: 10.1126/sciadv.abp8701 (PMC9524841; doi:10.1126/sciadv.abp8701)
Supplement: Supplementary file 1 — Figs. S1 to S10 [file sciadv.abp8701_sm.pdf]

Science Advances
AAAS

Supplementary Materials for

Ca^{2+} channels couple spiking to mitochondrial metabolism in substantia nigra dopaminergic neurons

Enrico Zampese *et al.*

Corresponding author: D. James Surmeier, j-surmeier@northwestern.edu

Sci. Adv. **8**, eabp8701 (2022)
DOI: 10.1126/sciadv.abp8701

This PDF file includes:

Figs. S1 to S10

Fig. S1.

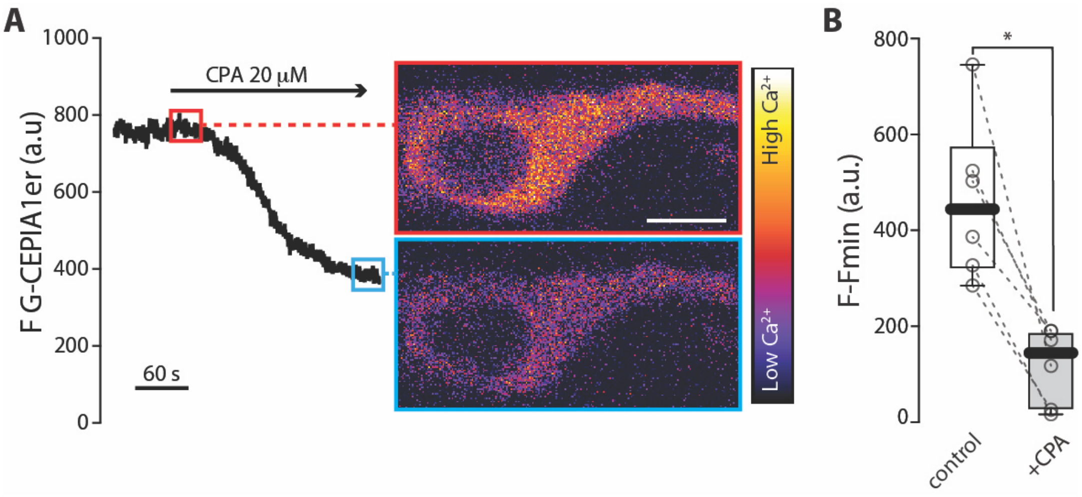

Detection of ER Ca^{2+} depletion induced by application of CPA with G-CEPIA1er.
(**A**) Representative 2PLSM G-CEPIA1er experiment showing depletion of ER Ca^{2+} upon application of the SERCA inhibitor cyclopiazonic acid (CPA); insets show representative 2PLSM images of the relative intensity of GCEPIA1-er fluorescence at different time points visualized with a look-up table (scale bar 10 μm). (**B**) Box-plots summarizing the effect of CPA on ER Ca^{2+} (n=6, N=5; P= 0.0156 1-tailed Wilcoxon matched-pairs signed rank test). Box-plots indicate first and third quartiles, thick center lines represent medians and whiskers indicate the range. a.u., arbitrary units. *P<0.05.

Fig. S2.

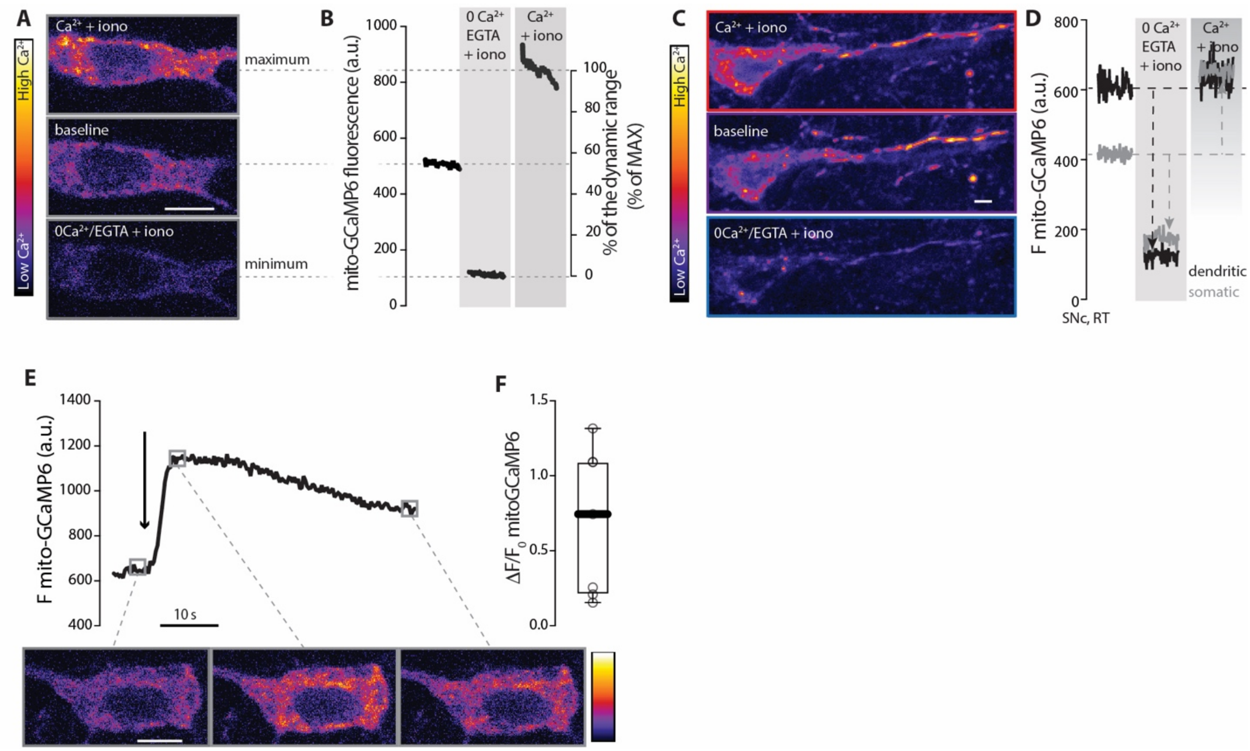

Representative 2PLSM mito-GCaMP6 experiments.

(**A**) 2PLSM images of a SNc dopaminergic neuron expressing mito-GCaMP6 during different steps of the calibration protocol: baseline, Ca^{2+}-free aCSF + ionomycin (iono, 1 μM), Ca^{2+} aCSF + ionomycin. (**B**) Representative 2PLSM time-lapse series indicating the mito-GCaMP6 fluorescence recorded at the different steps of the calibration protocol and expressed as a percentage of the dynamic range. (**C**) Representative 2PLSM images of somatic and dendritic mitochondria in a SNc dopaminergic neuron expressing mito-GCaMP6 during the different steps of the calibration protocol. (**D**) Representative 2PLSM time-lapse series indicating the mito-GCaMP6 fluorescence recorded at the different steps of the calibration protocol for somatic (gray) and dendritic (black) mitochondria. Note that experiments comparing somatic and dendritic mitochondria were performed at room temperature (RT). (**E**) Representative 2PLSM mito-GCaMP6 experiment showing mitochondrial Ca^{2+} uptake upon stimulation with mGluR-I agonist DHPG (black arrow, 10 μM) and representative 2PLSM images of the relative mito-GCaMP6 fluorescence intensity at different time points. (**F**) Box-plots summarizing the increase in mito-GCaMP6 signal induced by application of DHPG (n=7, N=7). Relative intensity of mito-GCaMP6 fluorescence in panel (**A**), (**C**) and (**E**) is visualized with a look-up table; scale bars in all insets: 10 μm. Box-plots indicate first and third quartiles, thick center lines represent medians and whiskers indicate the range.

Fig. S3.

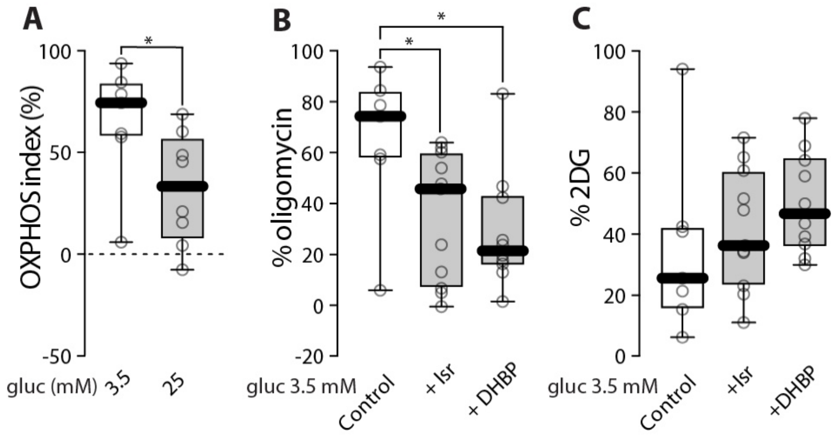

Mitochondrial contribution to SNc dopaminergic neurons bioenergetics in different conditions.

(**A**) Box-plots summarizing the different contribution of mitochondria to bioenergetics of SNc neurons in high and physiological glucose concentration: at physiological glucose concentration (3.5 mM) mitochondria oxidative phosphorylation is the source of ~ 70% of cellular ATP, versus ~ 34% at higher glucose (25 mM; P=0.0401 2-tailed Mann-Whitney test, n=7, N=6 and n=8, N=5 for 3.5 and 25 mM glucose). (**B-C**) Application of isradipine (Isr) or 1,1'-diheptyl-4,4'-bipyridinium dibromide (DHBP) specifically diminishes mitochondrial contribution to bioenergetics; box-plots showing the percentage of ATP/ADP ratio sensitive to oligomycin in different conditions (control vs Isr p=0.0393; control vs DHBP; p=0.0158 Dunnett's multiple comparison test, respectively; n= 7, N=6; n=11, N=8; n=10, N=6 for control, isradipine- and DHBP-treated respectively). Box-plots indicate first and third quartiles, thick center lines represent medians and whiskers indicate the range. *P<0.05.

Fig. S4

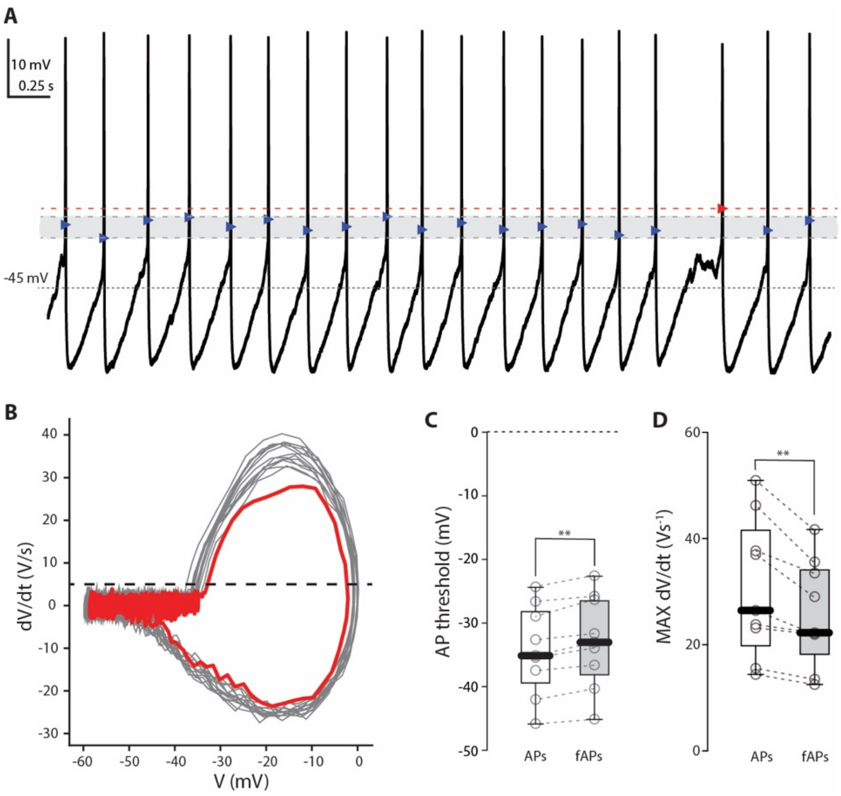

Electrophysiological characterization of changes in action potentials during failure events. (**A**) Representative recording of a SNc neuron at the onset of failure during stimulation (50 pA). The blue arrowheads indicate the action potential (AP) thresholds for the regular APs (the gray area highlights their range), the red arrowhead indicates the AP threshold for the failing AP (fAP). (**B**) dV/dt vs V phase plane plots of the APs in the recording in (**A**): in gray are the phase plots for the healthy APs, in red the one for the failing AP, which is shifted towards less negative V values and reach a lower maximum dV/dt. (**C**) Box-plots comparing the median threshold of healthy APs with the median threshold of failing APs. The fAPs have a higher (more positive) threshold (median of differences: 1.46 mV; 2-tailed Wilcoxon matched-pairs signed rank test P=0.0039, n=9, N=9). (**D**) Box-plots comparing the median MAX dV/dt of healthy APs and failing APs; fAPs have a decreased MAX dV/dt (median of differences: -4.22 V/s; 2-tailed Wilcoxon matched-pairs signed rank test P=0.0039, n=9, N=9). The recording at which each cell started failing was used for the analysis, independently of treatment or intensity of stimulation required to induce failure; control and isradipine treated cells are pooled together. Box-plots indicate first and third quartiles, thick center lines represent medians and whiskers indicate the range. **P<0.01.

Fig. S5

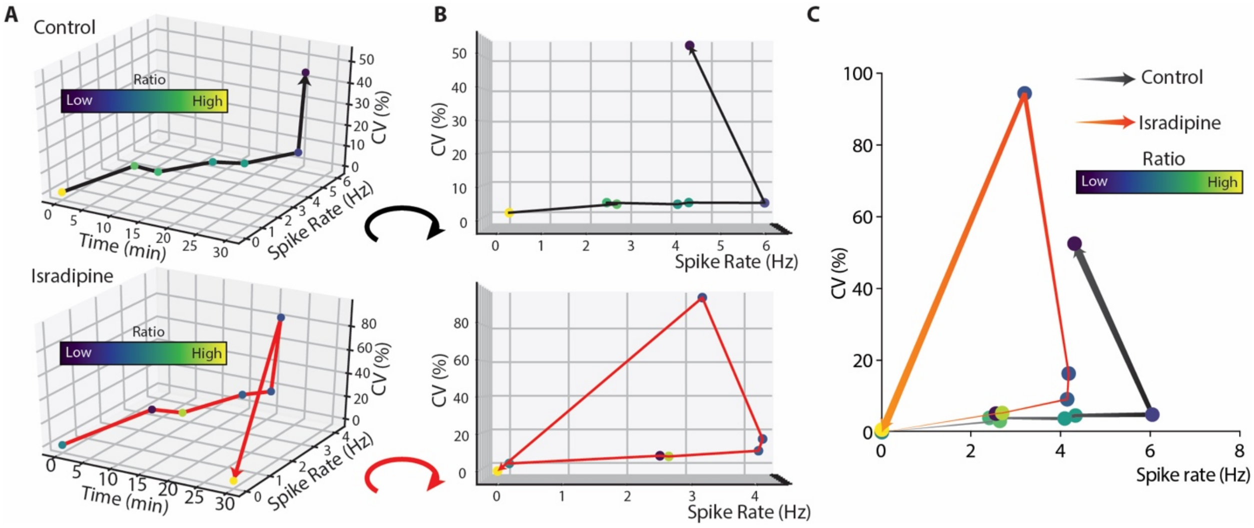

Variation of CV and PercevalHR ratio with spike rate over time in control and isradipine-treated cells. Detailed description of the generation of the plot depicted in Fig. 5F.

(**A**) 3D plots representing changes in PercevalHR ratio (as indicated by the color gradient) and coefficient of variation (CV, y axis) for different spike rates (z axis) over time (x axis), for representative control (top) and isradipine-treated (bottom) dopaminergic neurons expressing PercevalHR during perforated patch recordings. The colorimetric scale indicates the maximum (yellow), the minimum (dark purple) and the intermediate ratio values recorded within each experiment. (**B**) Axial rotation of the plots highlights how PercevalHR ratio and CV change upon changes in spike rate (time-axis is collapsed). (**C**) Combined plots of CV and color-coded PercevalHR ratio over spike rate for control (black) and isradipine-treated (red) neurons. The same plot is presented adopting a logarithmic scale for the y-axis (CV) in **Fig. 5F**.

Fig. S6

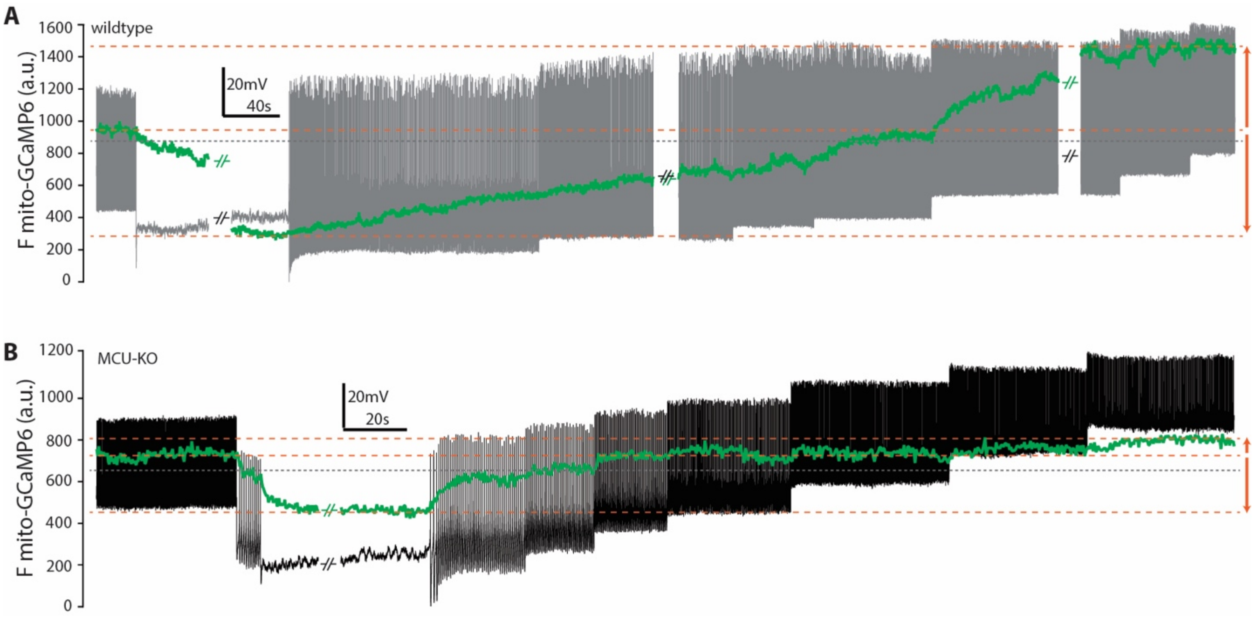

Representative mito-GCaMP6 traces in wildtype and MCU-KO dopaminergic neurons during changes in firing.

(**A, B**) Representative 2PLSM imaging experiments combined with perforated patch current clamp recordings in wildtype and MCU-KO neurons. (**A**) Wildtype neurons show large changes in mitoGCaMP6 fluorescence (green) upon changes in firing frequency. (**B**) MCU-KO neurons show a small drop in mitoGCaMP6 fluorescence (green) when firing is stopped, and minimal changes during increases in firing frequency. Gray dashed line: -40 mV.

Fig. S7

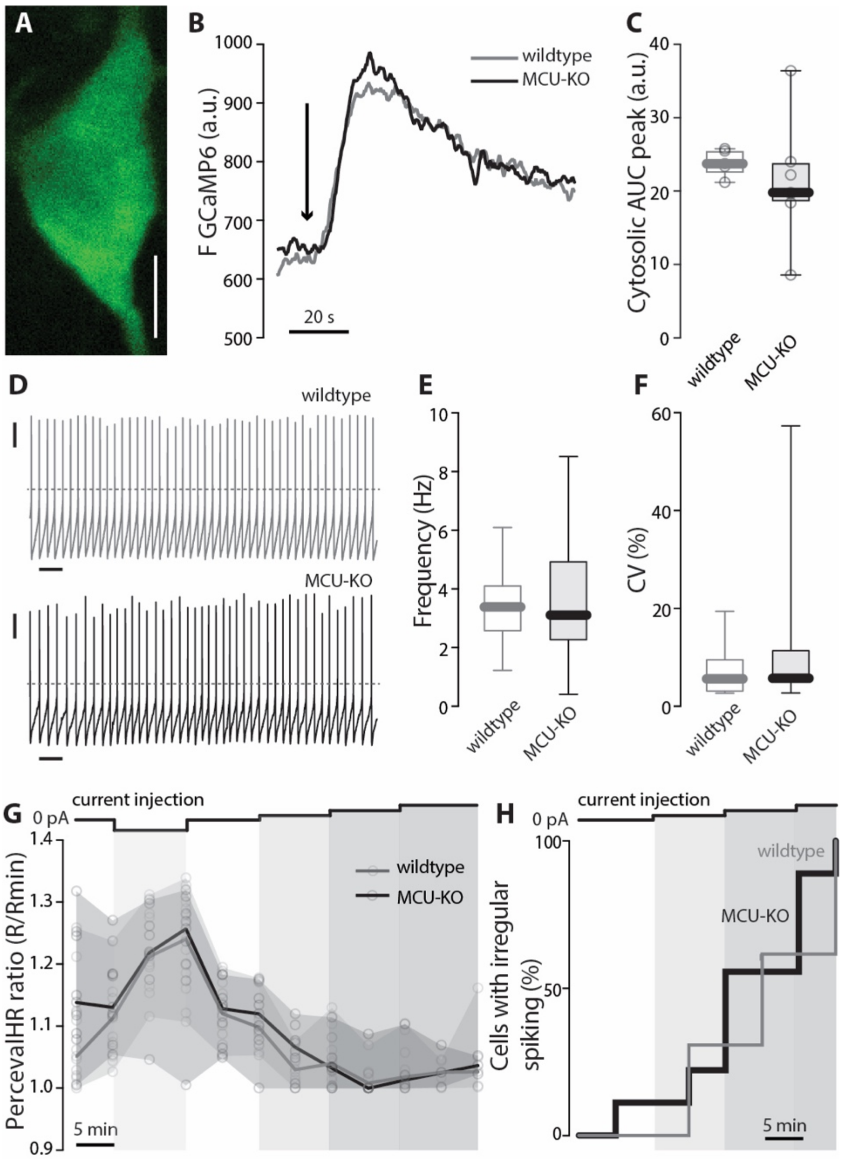

Cytosolic Ca^{2+} release, baseline and stimulated firing in MCU-KO SNc dopaminergic neurons.

(**A**) Representative 2PLSM image of a SNc dopaminergic neuron expressing GCaMP6s (scale bar 10 μm). (**B**) Representative traces of 2PLSM GCaMP6s experiments during stimulation with DHPG (black arrow, 10 μM) in wildtype and MCU-KO neurons. (**C**) Quantification of cytosolic Ca^{2+} release stimulated by DHPG in wildtype and MCU-KO neurons expressing GCaMP6s; the area under the curve (AUC) is calculated over 90 s from the start of the peak (n=5, N=4 for wildtype; n=7, N=4 for MCU-KO). (**D**) Representative perforated patch current clamp recording from wildtype (top) and MCU-KO (bottom) SNc neurons during baseline firing (scale bars: 10 mV, 1 s; dashed gray lines = -40 mV). (**E, F**) Box-plots summarizing baseline spike rate and

coefficient of variation in wildtype and MCU-KO neurons (n= 30, N=21; n= 33, N=22 respectively). (**G**) 2PLSM PercevalHR ratio imaging and perforated patch experiments in SNc neurons from wildtype (gray) and MCU-KO (black) mice during injection of negative and positive currents (medians, range and individual points are depicted; n=13, N=12; n=9, N=9 for wildtype and MCU-KO respectively). (**H**) Cumulative probability plots of cells undergoing failure to sustain firing at different time points for wildtype (gray) and MCU-KO (black) SNc dopaminergic neurons during the current injection protocol described in panel (**G**) (n=13, N=12; n=9, N=9 for wildtype and MCU-KO respectively). Box-plots indicate first and third quartiles, thick center lines represent medians and whiskers indicate the range.

Fig. S8

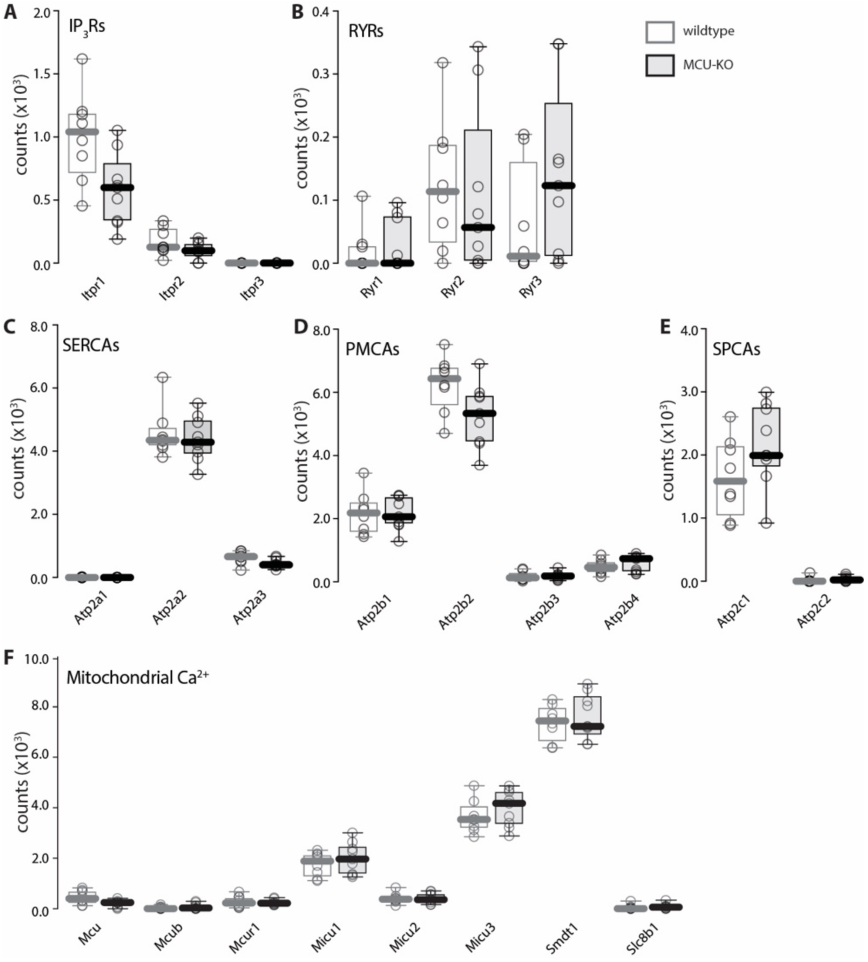

The essential Ca^{2+} homeostasis toolkit is not affected in MCU-KO dopaminergic neurons. **(A)** Expression levels analyzed by RNASeq of IP_3 Receptor (IP_3R) isoforms. (**B**) Expression levels analyzed by RNASeq of Ryanodine Receptors (RYR) isoforms. (**C**) expression levels analyzed by RNASeq of Sarco-Endoplasmic Reticulum Ca^{2+}-ATPase (SERCA) isoforms. (**D**) Expression levels analyzed by RNASeq of Plama Membrane Ca^{2+} Pump (PMCA) isoforms. (**E**) Expression levels analyzed by RNASeq of Secretory Pathway Ca^{2+} ATPase (SPCA) isoforms. (**F**) Expression levels analyzed by RNASeq of components of the mitochondrial Ca^{2+} Uniporter (MCU) complex and of the mitochondrial Sodium/Lithium/Ca^{2+} exchanger (NCLX, Slc8b1). Panels **A-F**: N=8, N=9 for wildtype and MCU-KO respectively. Box-plots indicate first and third quartiles, thick center lines represent medians and whiskers indicate the range.

Fig. S9

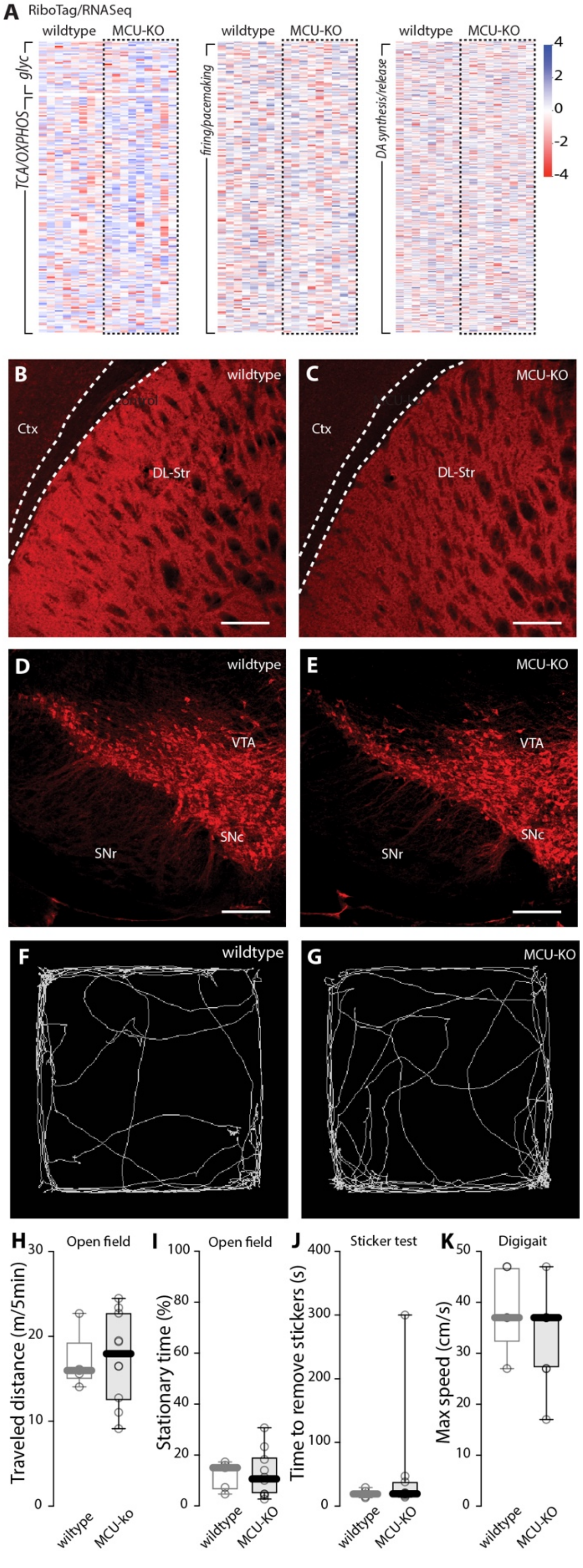
A RiboTag/RNASeq
wildtype
MCU-KO
glyc
TCA/OXPHOS
firing/pacemaking
DA synthesis/release
4
2
0
-2
-4
B wildtype
Ctx
DL-Str
C MCU-KO
D wildtype
VTA
SNc
SNr
E MCU-KO
F wildtype
G MCU-KO
H Open field
Traveled distance (m/5min)
I Open field
Stationary time (%)
J Sticker test
Time to remove stickers (s)
K Digigait
Max speed (cm/s)
wiltype
wildtype
MCU-ko
MCU-KO

Lack of histological and behavioral phenotype in MCU-KO mice

(**A**) Heat-maps of differential expression of genes linked to TCA cycle, OXPHOS and glycolysis, neuronal firing and pacemaking, and DA neurotransmission obtained from RNAseq analysis from wildtype and MCU-KO SNc neurons expressing RiboTag (N=8 and 9 for wildtype and MCU-KO). (**B-E**) TH immunostaining in dorso-lateral striatum (DL-Str, **B, C**) and in midbrain (**D, E**) does not show alterations in MCU-KO versus wildtype mice; scale bars = 100 μm (Ctx, cortex; SNr, substantia nigra pars reticulata). (**F, G**) Representative open field tracks for wildtype and MCU-KO mice. (**H**) Box-plots summarizing the traveled distance measured in the open field test over 5 minutes for wildtype and MCU-KO mice. (**I**) Box-plots summarizing the stationary time measured during the open field test over 5 minutes for wildtype and MCU-KO mice. (**J**) Box-plots summarizing the time required to remove the stickers for wildtype and MCU-KO mice. (**K**) Box-plots summarizing the maximum speed reached by wildtype and MCU-KO mice during the DigiGait test. (**B-K)** N=5, N=10 for wildtype and MCU-KO mice. Box-plots indicate first and third quartiles, thick center lines represent medians and whiskers indicate the range.

Fig. S10

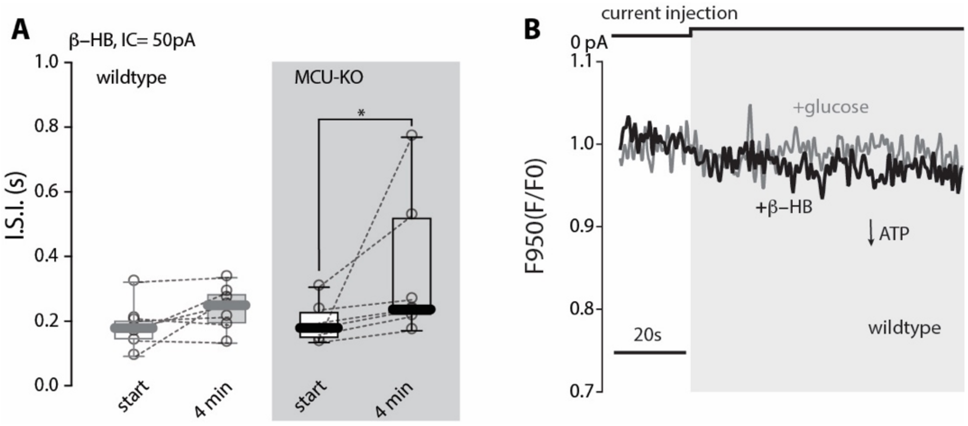

Change in spike rate during stimulation in beta-HB aCSF.

(**A**) Box-plots summarizing the change in inter-spike interval (I.S.I.) during perforated-patch recordings in beta-HB aCSF at the onset of the stimulation (50 pA) and at the end of the recording (4 min) for wildtype and MCU-KO neurons (wildtype, n=7, N=7; MCU-KO, n=7, N=7, P=0.0156 2-tailed Wilcoxon matched-pairs rank test). (**B**) Representative PercevalHR F950 traces for wildtype SNc neurons in glucose (gray) and beta-HB aCSF (β-HB, black) at the application of 50 pA stimulation. Box-plots indicate first and third quartiles, thick center lines represent medians and whiskers indicate the range. *P<0.05.
